# Vegetarian ethnic foods of South India: review on the influence of traditional knowledge

**DOI:** 10.1186/s42779-022-00156-1

**Published:** 2022-10-21

**Authors:** Srinidhi K. Parthasarathi, Ananda Vardhan Hebbani, Padma Priya Dharmavaram Desai

**Affiliations:** 1 Department of Management Studies, IA School of Management Studies, Bengaluru, India; 2 Department of Biochemistry, Indian Academy Degree College (Autonomous), Bengaluru, India; 3 Department of Basic Sciences, New Horizon College of Engineering, Bengaluru, India

**Keywords:** South India, Hinduism, Vegetarian ethnic foods, Philosophical perspective

## Abstract

South Indian cultures are diverse and unique amongst Indian traditions. In spite of many changes in Indian traditions over generations, South Indian states seem to have maintained a great extent of similarity with reference to vegetarian ethnic food habits and the reason behind is not convincingly known. Hindu traditional texts have extensive mention of the ethnic origins of many cultural practices prevailing in India and the present review aims to explore the different vegetarian ethnic foods of South India and also look into the influential role of food related ideologies mentioned in the traditional texts. Ethnographic study data about the prevailing vegetarian foods of the states were generated using multiple tools and presented. It is observed that there is a great extent of similarity amongst the varieties of vegetarian foods being prepared in Hindu communities of South India. Our study also highlights the strong influential role of tradition in evolution of vegetarian foods prevailing even today in South India.

**Supplementary Information:**

The online version contains supplementary material available at 10.1186/s42779-022-00156-1.

## Introduction

South India represented by five major Indian states viz., Tamil Nadu (TN), Karnataka (KA), Kerala (KL), Andhra Pradesh (AP) and Telangana (TG); is well known for its rich cultural heritage and food is one of the major contributors to its richness. Traditions and food are highly inter-influential throughout India, more so in South India. Based on the historical evidences, it is clear that the Dravidian civilization of South India is much more primitive (flourished approximately 4500 years ago) in comparison to the rest of Indian civilizations [1] and South Indian cuisines seemingly continue to retain many of the ancient Dravidian food culture elements.

According to India State of Forest Report (IFSR 2021), South Indian states (total geographical area of 6,35,748 km^2^) comprises of a high percentage of forest cover viz., 20.31% in TN, 20.19% in KA, 54.7% in KL, 18.28% in AP and 18.93% in TG; which enriches the food heritage of South India [2]. The states are also major contributors of agricultural economy of India and nearly 48% of its population engage in agriculture. Paddy, sorghum, pearl millet, pulses, chilli and ragi are the major crops cultivated in the South India. The South Indian states are also predominant spice-producing Indian states [3] and thus cuisines here are relatively more flavoured and spicy when compared to other Indian cuisines because of the extensive usage of spices such as ginger, paprika, coriander, cinnamon, tamarind, pepper and cumin seeds [4]. With reference to the dietary practices, reports show that approximately 30% of South Indian people are vegetarians [5] and they generally belong to either of the Brahmin, Arya Vysya, Lingayat and Nambudiri communities under Hinduism.

South Indian vegetarian cuisines seem to be unaffected by other cultural influences and people continue to prefer eating foods in the old traditional ways. Rice is the predominant component of a typical South Indian vegetarian meal since it is grown in large quantities in all the states of South India and people over generations are evolved with a natural affinity for rice. Moreover, since climate of South India is generally hot and humid throughout the year, it becomes congenial for assimilation and digestion of rice based foods. Additionally, a rich repertoire of ingredients and additives used for rest of the South Indian dishes viz., vegetables (both raw form or cooked), cereals, pulses, tamarind juice, curds, jaggery etc., makes the food more of a functional neutraceutical which is a unique feature of South Indian vegetarian cuisine. Thorough observation of the similarity that exists between various vegetarian South Indian dishes across the states (both in terms of the used ingredients or preparations procedures) clearly gives a clue about a sort of binding phenomenon behind their evolution. There is an increased necessity for understanding the ethnic origins of foods through a traditional perspective as it helps in knowing the population better. This ultimately would help in developing more practical healthcare regimens through scientifically relevant dietary modifications. Literature clearly suggests that traditional knowledge systems serve as strong repositories for understanding the evolutionary lineage of any age old practice [6] and this review attempts to understand the same especially for the vegetarian ethnic food practices of South India.

## Review of literature

Globally, there has been a consistent rise in ethnic food research, because of the growing demand for traditional foods and also because of the increased opinion that ethnic foods are the best sustainable alternatives to provide healthy food to the world’s population in future [7]. Indian ethnic foods are highly diverse and research clearly reveals that they are strongly influenced by culture, religion and traditional knowledge systems like Ayurveda [6, 8–11]. It is also known that many of the food ingredients used in Indian diets (viz., rice, salt, sugar, jaggery, mustard, turmeric etc.) are being mentioned in many pre-historic traditional texts like Vedas, Upanishads, Bhagavad Gita and Mahabharata [12]. There has been a considerable amount of literature available predominantly highlighting the ethnic fermented foods and beverages of various parts of North, East and West India and the key observations are being summarized in Table 1. Similarly South Indian fermented foods and beverages with specific ethnic names are elaborated in Table 2. Additionally non-fermented ethnic food cultures of Sikkim [25] and Chhattisgarh [26] are also being exclusively studied and presented. Thorough overview of existing studies on ethnic foods in India clearly indicates a scope to additionally understand Indian ethnic food culture from a traditional perspective, so that the influential role of traditional knowledge on the food practices could be established. There is not much of a work done with this objective and the present work is an attempt to understand the same particularly for the vegetarian ethnic foods of South India.

**Table 1:** Fermented ethnic food categories of North, East and West India

Title	Process involved	Food category (summarized from all the studies)	References
Ethnic fermented foods and beverages of India: science history and culture	Foods and beverages derived from natural fermentation	Fermented • Rice, legumes and cereal foods • Milk foods • Non-soy bean legume foods • Rice-legume mixture foods • Soy-bean foods • Bamboo shoot foods • Vegetable foods • Sundried/smoked fish products • Sundried/smoked meat products • Beverages • Tea/crabs/fruits etc	[13]
An overview on ethnic fermented food and beverages of India: Interplay of microbes, immunity and nutrition			[14]
Diversity of traditional and fermented foods of the Seven Sister states of India and their nutritional and nutraceutical potential: a review			[15]
Folk to functional: an explorative overview of rice-based fermented foods and beverages in India			[16]
Naturally fermented ethnic soybean foods of India			[17]
Traditional Indian fermented foods: a rich source of lactic acid bacteria			[18]
Dietary culture and antiquity of the Himalayan fermented foods and alcoholic fermented beverages			[19]
Fermented foods and beverages of Mizoram			[20]

**Table 2:** Fermented ethnic food items prepared in South India

Title	Regional area	Process involved	Food items	References
Ethnic fermented foods and beverages of Tamil Nadu	Tamil Nadu	Foods and beverages derived from natural fermentation	• Fermented rice (pazhayasadham) • Idli • Dosa/Dosai • Uthappam • Appam • Koozh • Dahi/Thayir • Dahi rice/Thayir sadham • Moru/Butter milk • Kallu/Toddy	[13, 21, 22]
Ethnic fermented foods and beverages of Karnataka	Karnataka		• Idli • Dosa • Adai Dosa • Sannas • Ambali • Pickle • Sandigie • Dahi • Butter milk • Ginna • Lassi • Neera • Palm Vinegar • Toddy • Cashew apple wine • Jamun wine • Kanji • Wine	[13, 23]
Ethnic fermented foods and beverages of Telangana and Andhra Pradesh	Telangana and Andhra Pradesh		• Idli • Dosa • Uttapam • Vada • Ambali • Taravani/kali • Dahi • Butter milk • Toddy/Kallu	[13, 24]

## Materials and Methods

South Indian vegetarian traditional preparations (especially served during a typical meal), their names across all the South Indian states, their brief preparation procedures and health benefits are being reviewed and presented. Additionally the food related ideologies (either in the form of sayings, precepts and prescriptions) mentioned in some predominant Indian traditional texts with a particular interest on ideologies presented by South Indian philosophers and theologians is also being discussed to establish the envisaged connect between traditions and practices.

### Study design, data sources and collection process

**Fig. 1:**
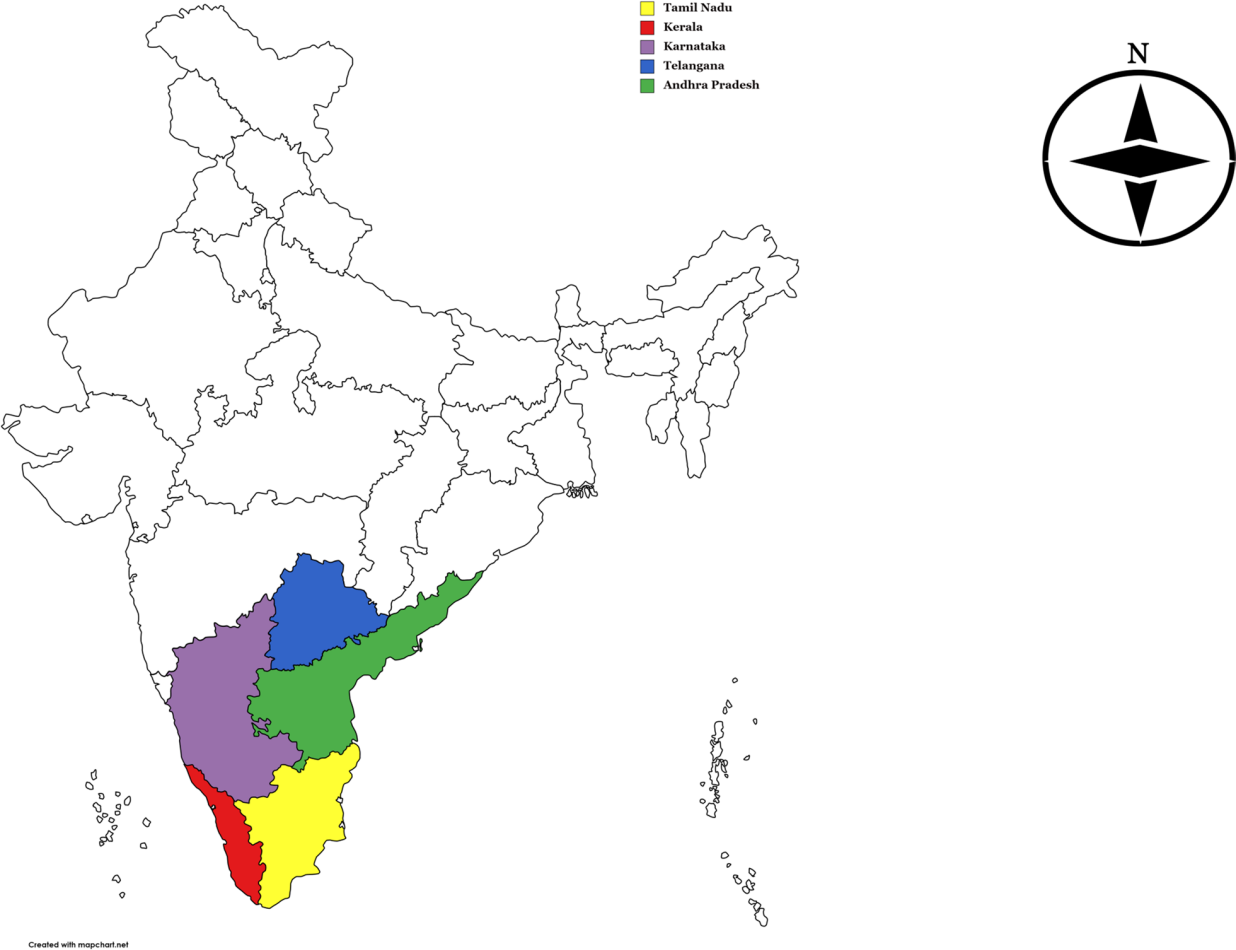
Geographical location of South Indian states

To understand food culture of the five South Indian states (Fig. 1), qualitative ethnographic studies were conducted as per standard fieldwork procedures using various methods viz., semi-structured interviews, direct observations and informal conversations [27]. Since the sources of generating authentic information for the present study is very limited (accounting to the fact that a mere 30% of South Indian population are vegetarians), studies were designed to cover all the prominent locations of all the five states where the ethnic groups of Brahmins, Arya Vysyas, Lingayats and Nambudiris would be concentrated and also living in the same locality from atleast 4–5 generations. Additionally, since most of the South Indian vegetarian foods are associated with one or the other religious activities, where in they are offered to god (as *prasadam*), religious heads and priests at the Hindu temples were also interviewed informally during the study.

Empirical evidences were generated by direct observations, which were conducted in-between October 2020 and November 2021. While many semi-structured interviews and informal conversations were initially done over phone during the above mentioned period (keeping in view the state wise restrictions during COVID 19), physical informal conversations and semi-structured interviews by site visits were conducted between November 2021 and February 2022.

To ensure privacy and comfort for the participants, all the semi-structured and informal conversations were conducted with their voluntary consent and at their leisure times either at homes or temples (when there wouldn’t be much crowd). The questions were basically to know the religious traditions being practiced in their families (Q1), their food preferences and preparation procedures (Q2) and foods being offered (*prasadam*) to god before consumption (Q3). In the case of interviews conducted at temples Q1 was to know the religious tradition being followed in the temple and Q2 was to know the foods being prepared as an offering to God.

### Data analysis

Based on the demographic fact that 30% of South Indian population (TN—2.35%, 21.1%—KA, 3.00%—KL, 1.75%—AP and 1.30%—TG) were vegetarian, a sample size of 140 was fixed to generate a data with 95% confidence level, 5% margin of error and representing 10% of the total vegetarian population. A total of 142 (42% were male and 58% were female—mean age 58.2 years) responses were obtained through semi-structured interviews and informal conversations (Participant profiles presented in Additional file 1: Appendix 1). Direct observational studies that were conducted at multiple instances by all the authors prior to the onsite/online conversations gave a better comprehensive picture of the actual area to be subsequently concentrated to generate authentic data for this study. The resultant empirical data is being scrutinized and analysed using standard constant comparison method, inductive analysis and triangulation approaches by thorough recheck—revision of informal questions to be asked, result analysis and interpretation [28].

## Results and discussion

The empirical data for the religious traditions (Q1) of the respondents seem to be clearly falling into either of the Brahmin (Vaishnava, Smartha, Niyogi, Badaganadu, Madhwa and Havyak communities) Arya Vysya, Lingayath or Nambudiri (Nair) ethnic groups across the five different states. Results of the foods with vernacular names across the states and their overall preparation procedures (Q2) is being presented below.

**Fig. 2:**
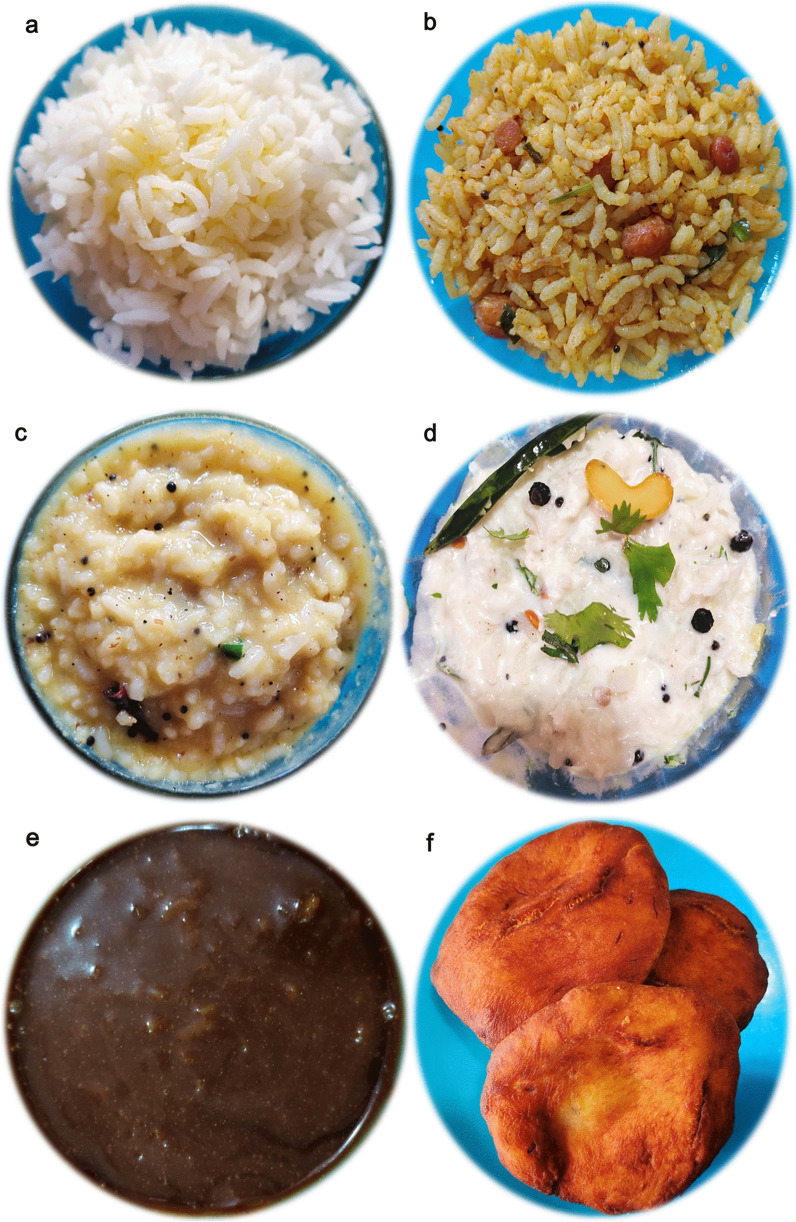

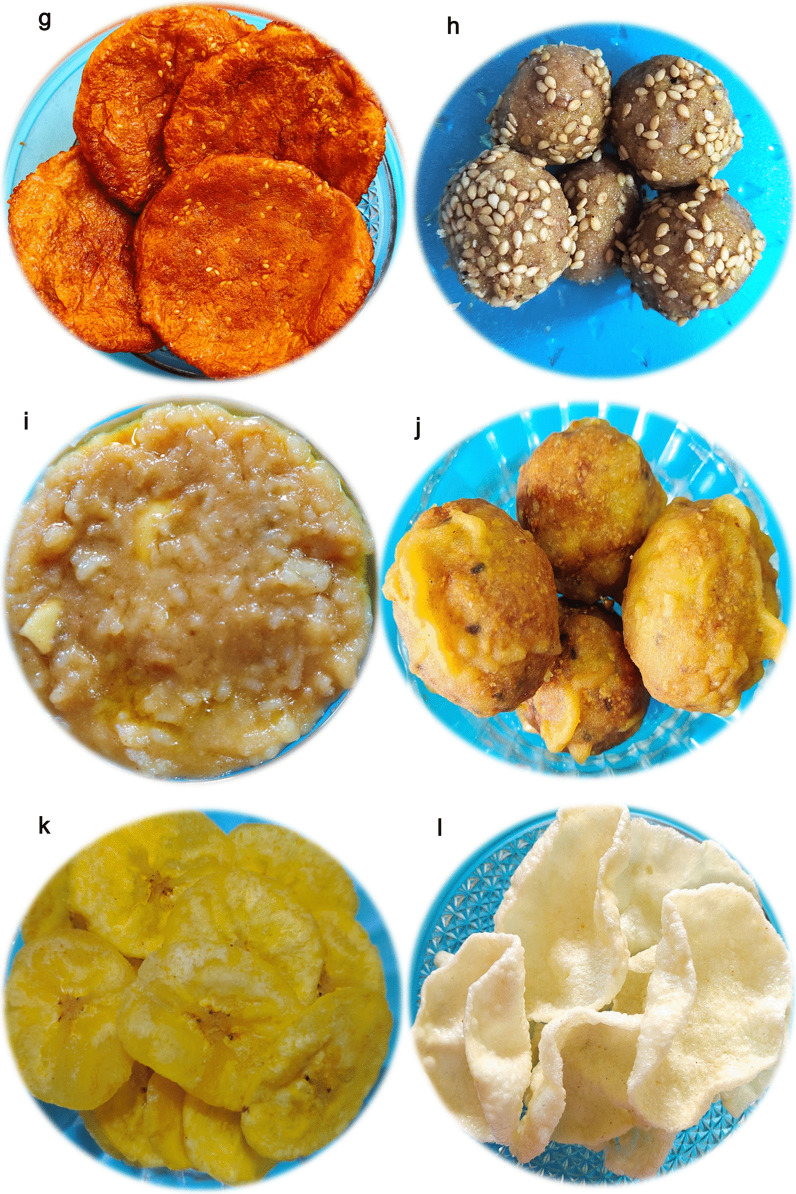

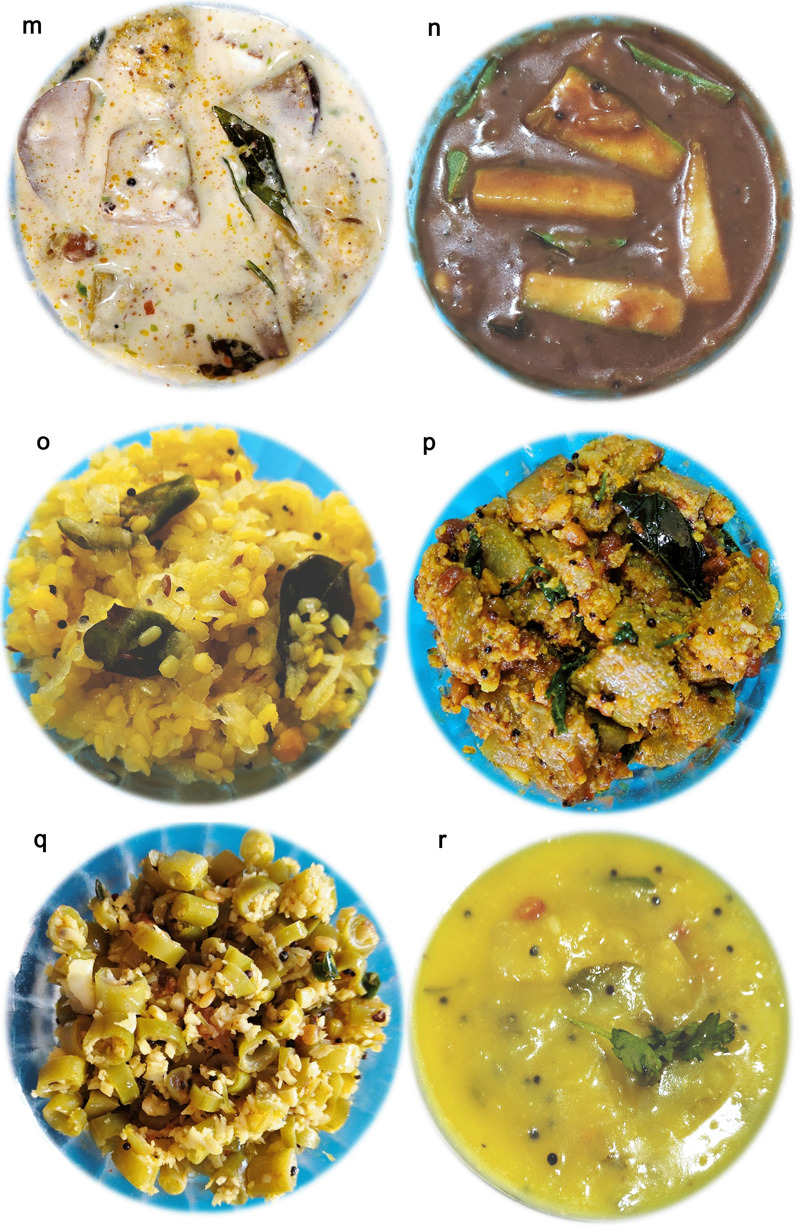

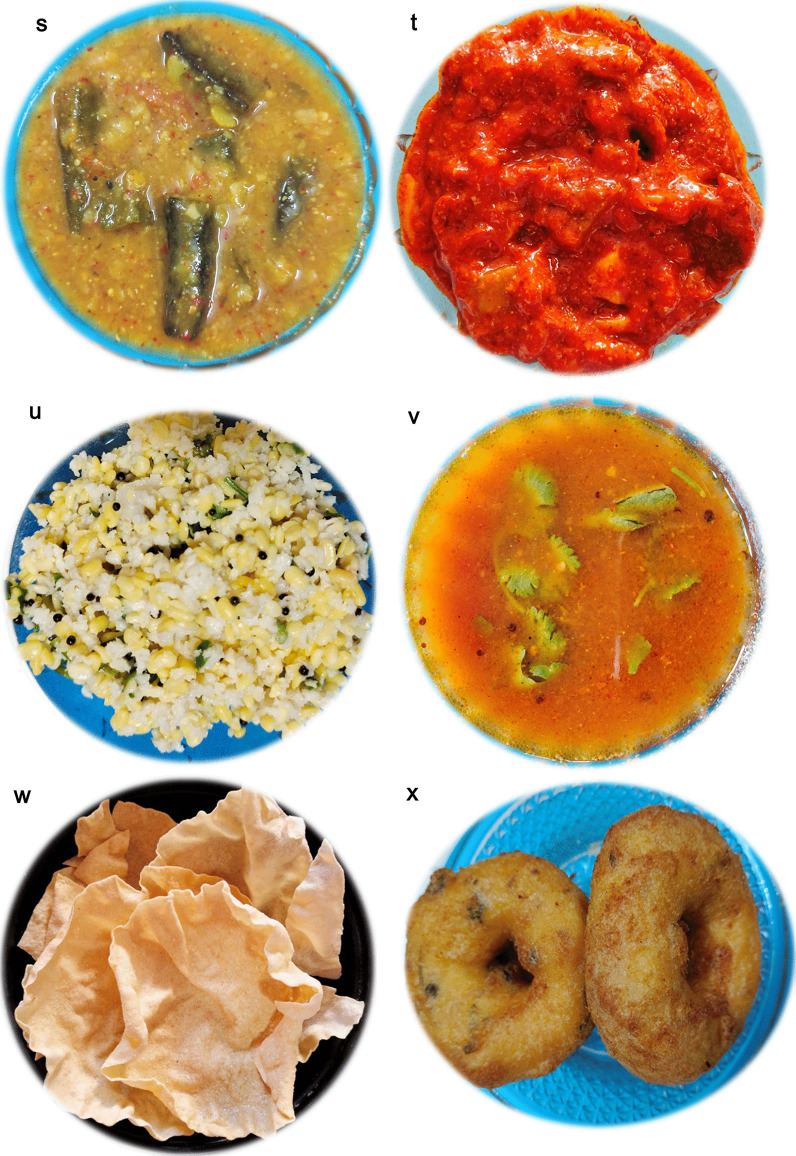

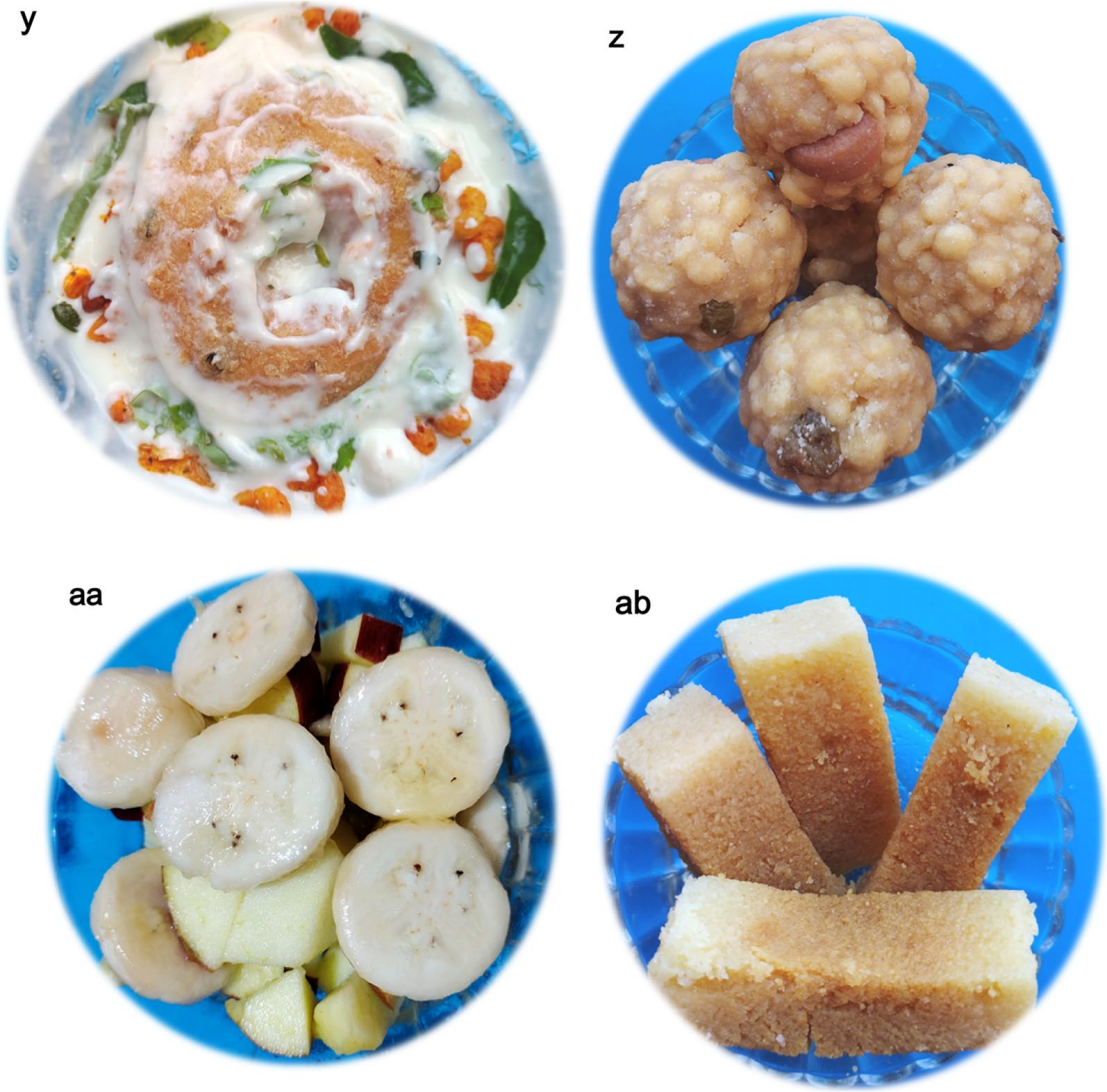
**a** *Nei sadam (TN)/Tuppada anna (KA)/Nei chorru (KL)/Neyyannam (TG, AP):* Apart from the medicinal values rice possess, ghee that is used in Nei sadam also possess many proven antioxidant and protective activities [31]. **b** *Puliyodharai or Puliyogharai (TN, KL)/Puliyogare (KA)/Pulihora (TG, AP):* Tamarind juice, the major ingredient of this dish, is proven to possess many health benefits especially aiding gastrointestinal tract for easy food processing [32]. **c** *Pongal (name continues in all five states):* Green lentils used in the dish are known sources of many phytochemicals conferring them with many medicinal properties [33]. Similarly cumin seeds and pepper corns are potent neutraceuticals which possess many medicinal values, more importantly antioxidant, antidiabetics and anti-inflammatory properties [34]. **d** *Dadhyodanam (name continues in all five states)*: Curd, the predominant ingredient of the dish is a known source of calcium and probiotics [35]. **e** *Tirukannamadai (or) Tirukannamadu (TN), Nei Payasam (KL)/Aravani (KA, AP)*: The dish possesses many health benefits since red rice is proven to have antioxidant, antidiabetic and antiproliferative activities [36–38]. Similarly molasses is also proven to possess antioxidant, anti obesity, anti microbial and anti cancer potentials [39]. **f** *Appam (TN, KA, TG, AP)/Unni Appam (KL)*: Apart from the medicinal values rice possesses, jaggery is proven to possess many micronutrients which have medicinal properties such as anticarcinogenicity and antitoxicity [40]. **g** *Atirasam (TN, KL)/Kajjaya (KA)/Ariselu (AP, TG)*: Apart from the medicinal values of rice and jaggery, sesame seeds topped on the dish are known to be rich in oils with high levels of unsaturated fatty acids and many other micronutrients, which confer many medicinal properties. Sesame oil is known to decrease lipid peroxidation and increase antioxidant status of the body when consumed [41]. **h** *Sidai (or) Seedai (TN)/Cheedai (KL, KA)/Undalu (TG, AP)*: Medicinal values of all the ingredients used in the dish are mentioned above. **i** *Tirukannamudu (TN)/Pal Payasam (KL)/Paramanna (KA)/Paramannam (TG, AP)*: The dry fruits used for garnishing the dish brings an additional neutraceutical property to the dish viz., cashew being rich in magnesium and calcium is known to support healthy muscles and gums, while raisins being rich in calcium and boron is known to aid in maintenance of bone, eye and dental health [42]. **j** *Sukhiyan (or) Sugiyan (TN, KL)/Sukkinunde (KA)/Purnalu (TG, AP)*: Green gram used in the dish is a protein rich resource with high dietary fibre, vitamin and mineral contents; which confer the ingredient with many medicinal values like antioxidant and hypolipidemic activities [43]. **k** *Varuval (TN)/Varavu (KL)/Vadiyam (KA, TG, AP)*: Raw banana is proven to possess higher amounts of functional ingredients such as dietary fibre and total starch, which impart better nutritive values in comparison to the ripened banana [44]. Similarly, jackfruit, being a rich resource of many vitamins, minerals and carotenoids in particular, is proved to confer protections against many chronic conditions including cancer, hypertension and coronary heart disease [45]. **l** *Vadam and Vatral (TN, KL), Sandige (KA), vadiyam (TG, AP):* Sago grains being very rich in carbohydrates, turns translucent and spongy after cooking, because of which it can become a base material like rice to prepare a snack and is preferred to be munched along with other spicy foods. **m** *Aviyal (TN, KL)/Majjigae Huli (KA)/Majjiiga Pulusu (TG, AP)*: Grated coconut predominates the taste while consuming Avial and is known to possess many health benefits like cardioprotection [46] and being high in fibre also aids weight loss and digestion health [47, 48]. **n** *Pulippu koottu (TN)/Puliserry (KL)/Gojju (KA, TG, AP):* While tamarind concentrates health benefits are already mentioned above, high mineral content of the gourd family vegetables is understood to confer significant prebiotic ability [49]. **o** *Poritta koottu (or) Poricha (TN, KL)/Playa (KA)/Vepudu (TG, AP)*: Along with the other ingredients for which the medicinal values are mentioned above, green legumes that are predominantly seen in the dish are well proven low-GI foods and are also hypocholesterolaemic [50]. **p** *Puli kariamudu (or) Puli Poriyal (TN, KL), Gojju (KA, TG, AP)*: The dish is semisolid in its consistency and is protein, fibre rich and thus very healthy. Due to its tangy sour taste because of tamarind concentrate, the dish is generally slurped in little quantities along with other foods. Health benefits of all the ingredients used in the dish is mentioned above. **q** *Kariamudu (or) Kuzhambu (TN)/Podduthol (KL)/Palya (KA)/Koora (TG, AP)*: The dish is generally dry and is also rich in proteins and fibres. Health benefits of all the ingredients used in the dish is mentioned above. **r** *Paruppu usili (TN), Parippu (KL), Bele playa (KA)/Koora (TG, AP)*: All the lentils are generally known to be rich resources of many bioactive peptides and thus have potential health benefits such as anticarcinogenic, hypocholesterolaemic and antidiabetic effects [51]. **s** *Kulumbu (or) Kolambu (TN)/Huli (KA)/Sambar (KL, TG, AP)*: Pigeon peas which make the predominant ingredient of Kulumbu is well proven not just as a protein and carbohydrate rich nutrient, but also highly medicinal due to the rich presence of multiple polyphenols and flavonoids. The phytochemicals of pigeon peas is proven to be anti-oxidant and anti-inflammatory in nature conferring many health benefits such as hepaprotective, hypoglycemic and even cancer prevention properties [52]. Moreover the major components used for seasoning are also highly therapeutic viz., coriander possessing antioxidant, anticonvulsant, antidiabetic and antihelmentic properties [53] and asafoetida which is antidiabetic, hypolipedimic, anti-helminthic, anti-metastatic, anti-diarrhoeal and even neuroprotective [54]. **t** *Oorugaai (TN)/Uppilittat (KL)/uppinakayi (KA)/Uragaya (TG, AP)*: Lactic acid bacteria contributing for the pickling process are also known to possess probiotics features and thus confer health benefits such as protection of GI tract from infections, prevention of urogenital infections, increasing digestion capacity, hypocholesterolaemic and suppression of cancer [55]. **u** *Paruppu Avial (or) Paruppu Avial (TN, KL)/Sundal (KA)/Guggillu (TG, AP):* The dish is a rich source of calories owing to the fact that the ingredients are proven to possess high nutritional value [56, 57]. **v** *Satramudu (or) rasam (or) chaaru (or) saaru in all five states:* Being rich in multiple ingredients the dish is proved to possess antipyretic, hypoglycaemic and antimicrobial properties. Also rasam is proven to be a best cure for anaemia and also reported to be of use in increasing lactation [58]. Spices used in rasam are also reported to possibly regulate immunity by interfering with many inflammatory factors and confer protection against COVID-19 disease [59]. Owing to its prophylactic immunobooster properties, there also had been a reported rise in number of outlets selling readymade rasam or rasam powder during peak times of COVID-19 infections worldwide. **w** *Appalam (TN)/Pappadam (KL)/Happala (KA)/Appadam (TG, AP)*: Lentils the major ingredient of the dish, are known protein rich source which is low on fat. They are proven to reduce the risks of diabetes, obesity and coronary heart disease [60]. **x** *Vadai (TN)*/*Vada (KA, KL, TG, AP):* Black gram, the major component of this dish is an established protein and dietary fibre rich food. Flour of black gram is enriched with multiple phenolic acids conferring anti-diabetic and antioxidant properties [61]. **y** *Tayir vadai (TN, KL)/Masuru vada (KA), Perugu Vada (TG, AP):* While the health benefits of black gram used in preparing vadai is already explained above, curds that are used additionally makes the dish a good neutraceutical rich functional food. **z** *Laddu (name continues in all five states)*: They are rich resources of carbohydrates, proteins, vitamins and unsaturated fatty acids; with many health benefits viz., cardioprotective, antidiabetic and anticancer potentials [62]. **aa** *Thaen palankal (TN, KL)/Rasayana (KA)/Rasavali (TG, AP)*: Ripened banana, the major fruit widely preferred for the salad is a rich resource of phenolics, carotenoids, flavonoids and biogenic amines which confer the fruit with multiple health benefits like antioxidant, antitumor, hypoglycaemic and hypocholesterolaemic activities. Also, being a rich source of potassium, iron, serotonin and vitamin A, banana becomes a wholesome food dessert ingredient in South Indian meals [63]. Additionally honey that is used as a topic on the dish is also a well proven to be rich resource of many flavonoids and phenolic acids which confer it with an array of health benefits [64]. **ab** *Tirattupal (TN)/Palkatti (KL)/Kova (KA, TG, AP)*: Milk is considered a complete food since it is rich in proteins, minerals like calcium and vitamins like A, B1, B2 and B12. While normal boiling of milk ensures its safety for human consumption and also offers many health benefits [65]; prolonged boiling alters the milks organoleptic features to a sweet and thick protein coagulated mass, which is a preferred form of milk for this dish. Moreover, addition of raw cardamom and pistachios makes the dish rich flavoured and healthy too, up to a limited quantity. Both cardamom and pistachios are proven to possess many health benefits. Cardamom is a major cultivated spice in South India and is known for its aroma due to the rich presence of volatile oils such as α-terpinyl acetate and 1,8-cineole. The oils also are proven to relieve conditions such as bronchitis, depression and other infections [66]. Similarly, pistachios having high levels of unsaturated fatty acids, potassium and tocopherols possess antioxidant and anti-inflammatory properties [67]

1. Rice based foods: Rice is the predominant item in any South Indian platter, which is generally cooked and used as a base for mixing and eating different varieties of other dishes ranging in tastes from bland to spicy to hot. Additionally rice based sweets are also prepared especially during festivals. Not just being a staple food, rice is also known to be a low fat diet; rich in carbohydrates, proteins, vitamins and minerals [29]. Moreover, apart from the general white rice, there exists an extent of flexibility with reference to the other varieties of rice (pigmented rice), with much more medicinal benefits, that are generally used in South Indian dishes [30]. The general improvisations done with rice and the resultant South Indian vegetarian foods are as follows
  - Plain rice and variants
    *Nei sadam:* Nei sadam is the main ingredient of any South Indian meal (Fig. 2a). Rice grains are cleaned and steam cooked to form a flowery white dish with great aroma. The dish is generally topped with ghee and consumed as such or along with other dishes.
    *Puliyodharai:* Puliyodharai is one of the frequently consumed dish both during festivals and on regular basis (Fig. 2b). Rice is cooked and mixed with gravy enriched with a thick seasoning of lentils, chillies, groundnuts, curry leaves, asafoetida, thick tamarind juice, jaggery and dried grated coconut..
    *Pongal:* Pongal is the most frequently prepared breakfast and is generally consumed steaming hot (Fig. 2c). It is prepared by cooking rice with green lentils and finally seasoned with generous amounts of cumin seeds, pepper corns, cashew nuts sautéed in ghee..
    *Dadhyodanam:* Dadhyodanam is special curd rice prepared on a regular basis and consumed at the end of a sumptuous meal (Fig. 2d). Cooked and slightly cooled rice is mixed with thick curds until a semisolid consistency and then seasoned to prepare the dish.
  - Rice based sweets
    *Tirukannamadai*: Tirukannamadai is a dessert prepared by sautéing red rice in ghee and finally mixed with molasses (Fig. 2e). The dish is generally preferred as a side dish for a meal especially during festivals.
    *Appam:* Appam is a sweet generally prepared during festivals or even eaten as an evening snack (Fig. 2f). Rice flour is mixed with jaggery syrup and kneaded to make a batter. Later, the batter is poured as small cakes on a pan and fried using ghee.
    *Atirasam*: Atirasam is a very traditional sweet generally prepared during festivals and composition wise it is a richer version of Appam mentioned above (Fig. 2g). For Atirasam, rice flour is mixed with jaggery to prepare dough with uniform consistency, which is rolled into round shaped cakes, sprinkled with sesame seeds and deep fried in ghee.
    *Sidai*: Sidai is prepared both as a sweet or a savory by using fried rice flour, either mixed with jaggery syrup or cumin seeds respectively (Fig. 2h). The mixture is rolled over lightly sauteed sesame seeds into balls and deep fried in oil/ghee.
    *Tirukannamudu*: Tirukannamudu is a dessert prepared to be served along with meals during festivals or even eaten regularly as an independent snack (Fig. 2i). The dish is prepared by boiling rice in milk along with jaggery and ghee. The dish is finally garnished by layering it with ghee fried cashew nuts and raisins.
    *Sukhiyan*: Sukhiyan is another traditional sweet prepared during festivals (Fig. 2j). Overnight soaked green gram is ground into a semi smooth viscous paste and ground jaggery is mixed with it and the kneaded paste is shaped into balls, dipped in rice batter and deep fried in ghee.
  - Rice based savouries:
    *Varuval*: Varuval is a dried side dish eaten along with rice mixed with spicy items (Fig. 2k). They are generally prepared and stored because of a reasonably good shelf life. Raw banana or yams or Jack fruit, are cut into fine slices, sun dried and then deep fried in oil and finally garnished with salt and dry chilli powder.
    *Vadam and Vatral:* Vadam and Vatral are also similarly used snacks as mentioned above (Fig. 2l). Rice flour or sago grains are cooked to a thick, paste like consistency and poured into small circles and dried under direct sunlight. Just before consumption the dried wafers are deep fried in oil.
2. Vegetable based foods
  - Vegetable based gravies and soups:
    *Aviyal*: Avial is a traditional south Indian dish prepared for all types of special festive occasions, generally eaten along with rice (Fig. 2m). The dish is prepared by using boiled vegetables mixed with grated coconut and thick curd making it a specially flavoured dish.
    *Pulippu koottu*: Pulippu kutu is a regularly prepared side dish generally preferred to be consumed along with rice (Fig. 2n). For the dish, gourd vegetables (bitter gourd, snake gourd, bottle gourd or ridge gourd) is cooked along with soaked tamarind concentrate and finally seasoned with lentils and red chillies to make a gravy.
    *Poritta koottu*: Poritta koottu is a relatively dry version of a side dish as mentioned above, generally consumed along with rice (Fig. 2o). Here, green legumes or gourd vegetables are mixed with lentils and grated coconut and the mixture is fried or sauteed to make the dish.
    *Puli kariamudu*: Puli kariamudu is also a regularly consumed side dish during a meal (Fig. 2p). Vegetables like okra, raw brinjal or raw banana is mixed with spices, cooked with tamarind concentrate and seasoned to prepare the dish.
    *Kariamudu*: Kariamudu is also a very frequently prepared side dish, generally consumed along with rice (Fig. 2q). Here, plain steamed vegetables are mixed with spices, fresh coconut and seasoned.
    *Paruppu usili*: Paaruppu usili is a very soft side dish prepared using lentils which is cooked and made into a paste and then mixed with cooked vegetable and seasoned (Fig. 2r). The dish is preferred to be consumed along with rice during a meal. Out of different varieties of lentils, traditional South Indian food recipes generally use green gram, black gram and horse gram to prepare Paruppu usili.
    *Kulumbu*: Kulumbu is an authenticated semi solid dish consumed with rice (Fig. 2s). Yellow split pigeon peas is cooked like a thick sauce and mixed with boiled/fried vegetables and further boiled with a powder (fried and ground lentils, red chillies, coriander seeds and dried coconut). The content is finally seasoned along with a sprinkle of asafoetida. The dish is a compulsory ingredient of a typical South Indian vegetarian meal and is generally preferred to be consumed hot.
  - Vegetable based fermented pickles:
    *Oorugaai*: Oorugaai are premade fermented pickles consumed as a side dish in very minute amounts, along with any rice mixed foods (Fig. 2t). Tingling nature of the dish is due to the pickling process that happens on the ingredients over time. Freshly cut ginger, mango or slices of lime are mixed with gingelly oil, turmeric, dry red chilli powder, fenugreek powder, mustard powder and salt. The mixture is stored in airtight porcelain jars for atleast 1–2 weeks and then consumed.
3. Other Cereal and Pulses based foods:
  - Cereal and Pulses based starters:
    *Paruppu Avial*: Paruppu Avial is a dish prepared with pre-soaked pulses boiled with salt and seasoned (Fig. 2u). Two varieties of pulses can be used for the preparation i.e., green gram or chick peas. Generally the dish is preferred either as a snack or a side dish during a meal, especially during festivals.
  - Cereal and Pulses based appetizer:
    *Satramudu*: Satramudu is considered a healthy appetising dish consumed along with rice or directly slurped like any typical soup (Fig. 2v). Thin and delicate supernatant on the cooked yellow split pigeon peas, is collected and boiled with spicy powdered mix of cumin seeds, fenugreek seeds and pepper corn. The mixture is finally seasoned along with a sprinkle of asafoetida. Rasam is considered as the most popular South Indian traditional foods and is considered very ideal recipe following the principles laid by Ayurveda, the Indian system of medicine.
  - Cereal and Pulses based savoury:
    *Appalam*: Appalam is also a similarly used snack as mentioned above (Fig. 2w). Dough of lentil flour is rolled into thin wafers and dried. Just before consumption the wafers are deep fried in oil.
    *Vadai*: Vadai is a widely and regularly consumed breakfast/lunch dish, generally eaten along with Idali—Dish 8 (Fig. 2x). Pre-soaked ground black gram is made into plain batter or with grated chillies and coconut and deep fried like a doughnut.
    *Tayir vadai:* Tayir Vadai is also a widely consumed breakfast/lunch item and it is a Vadai (Fig. 2y), post soaked in curds, garnished with curry leaves and coriander.
  - Cereal and Pulses based sweets:
    *Laddu*: Laddu is a ball shaped traditional sweet prepared during auspicious Hindu occasions (Fig. 2z). Gram flour is mixed with water to a semi liquid consistency and poured over a porous filter to form small sized droplets into boiling oil and deep fried. Handful size of fried and dried droplets are bonded together with thick sugar syrup and rolled into small balls. Garnishing of the balls with cloves, raisins and cashew nuts, gives an additional flavour and taste. Chickpea, the primary source to prepare gram flour is a vastly grown and consumed crop in Asia.
4. Fruit based foods
  *Thaen palankal*: Thaen palankal is fruit salad topped with honey or sugar (Fig. 2aa). The dish is frequently consumed along with a meal or as a supplement during rituals, when people do fasting.
5. Milk/milk product based foods
  *Tirattupal:* Tiruttupal is concentrated milk sweet consumed occasionally (Fig. 2ab). Cream milk is boiled for a prolonged period of time to reduce into a thick consistent gel, with a midway addition of sugar. The gel is poured into the required shape while hot and seasoned with a sprinkle of cardamom and slices of pistachios.

Responses also clearly indicate that the above mentioned foods had been in existence in the respondent families from the past four to five generations. Responses to Q3 clearly gives a hint that atleast 80% of the foods appear to be offered to god as *prasadam* (Data presented in Additional file 1: Appendix 1). Moreover, responses from the semi-structured interviews conducted at temples confirm that many foods prepared at temples are mostly the same across all the five different states, with only a vernacular difference of names. Results of the present study thus clearly gives a hint about the strong association between food practices and religious traditions across all the five South Indian states, which is further established by looking into the phenomenon of food according to Indian Hindu traditional knowledge system. Since origination of any food practise though predominantly are influenced by climatic conditions and availability of culinary ingredients, role of traditional beliefs is undeniable.

### Foods according to Indian Hindu traditional texts

The ancient Indian Hindu wisdom gives insightful references about different categories of food and their effects on human beings. Hindu philosophical knowledge, with its roots in the Vedas, is being perceived and branched out as six schools of philosophy viz. *Sankhya, Yoga, Nyaya, Vaisheshika, Mimamsa* and *Vedanta*. As per these six schools of philosophy, all knowable things are divided into two kinds ie. the means of knowledge (*Pramana*) and the object of knowledge (*Prameya*). The objects of knowledge are classified into substance (*Dravya*) and non-substance (*Adravya*). *Dravya*- the substance is further categorized as material (*Jada*) and immaterial (*Ajada*). *Jada*– the material component is formed by *Prakruthi* along with time, whereas the *Purusha* (individual soul) forms the immaterial component [68, 69]. The *Sankhya* system propounded by *Kapila* delineates that the creation is made of two interdependent realities, the *Prakruthi* and the *Purusha*. *Prakruthi* or the material component has been created with three innate dispositions viz., *Sattva* (Goodness), *Rajas* (passion or activity) and *Tamas* (darkness or inertia); which have their effect on all the materials or substances including food and mind [70, 71]. Foods can thus be classified into three categories viz. *Sattvik*, *Rajasic* and *Tamasic* foods and this categorization method also has a deep-rooted reference in the universally accepted song of the lord “*Bhagavad Gita*” verse *17.7* [72–74]. Literature suggests that *Sattvik* foods like vegetable, fruits, nuts and whole grains are non-irritating to the stomach and induce calmness and nobility to the person consuming by increasing energy of mind; *Rajasic* foods like meat, eggs, fish, spices, onions, garlic, hot peppers and pickles confer emotional, passionate, restless qualities like anger, delusion, fantasies and egotism; and *Tamasic* foods like leftovers, stale, overripe, spoiled foods are considered to produce negative emotions like pessimism, laziness and doubt [75]. As per *Chandogya Upanishad 6.5.4*, while quality of food which is being consumed is known to influence the mind, people with the innate dispositions of *Sattva*, *Rajas* and *Tamas,* seems to prefer the foods which are associated with their respective innate dispositions [76].

**Fig. 3:**
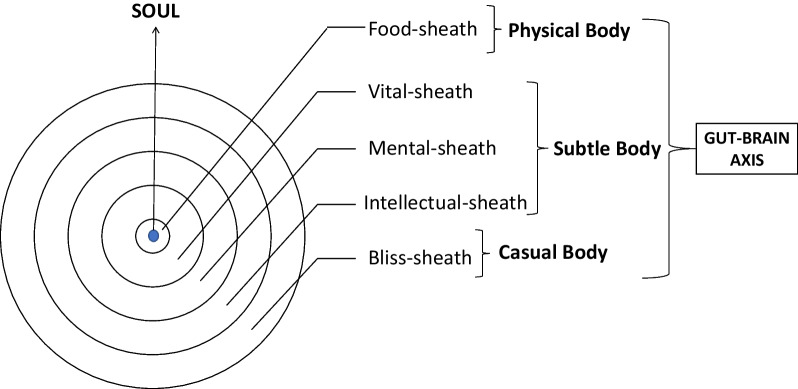
Layers around the soul and its relation with phenomenon of Gut-Brain Axis

Hindu philosophy clearly states that individual soul (*jiva*) is enclosed within five-sheaths termed the *Kosas* viz., food-sheath (*annamaya kosa*), vital-sheath (*pranamaya kosa*), mental-sheath (*manomaya kosa*), intellectual-sheath (*vijnanamaya kosa*) and the bliss-sheath (*anandamaya kosa*) [77]. This clearly appears to have a correlation with the modern phenomenon of gut-brain axis, wherein the food-sheath can physiologically be considered as the digestive system and the other sheaths would constitute the different neuroendocrinal components ultimately culminating at brain. The phenomenon is elaborated for the ease of understanding in Fig. 3. According to Paramahamsa Yogananda, the famous Indian theologian and yoga guru; diet affects the consumers psychological disposition, as it has a bearing on his/her state of mind both favourably and unfavourably. It is also being said that the food one consumes has a relationship with the mind and thus eating the right kind of food is a must for maintaining a healthy body and brain [78]. Since the dispositions of mind are believed to have got by their past experiences which cause impressions called *vasanas,* a person with innate disposition (*vasana*) of *Sattva* prefers to have *saatvic* food, a person with *Rajas* innate disposition prefers *rajasic* food and a person with a innate disposition of *Tamas* would prefer *Tamasic* food—*Bhagavad Gita 17.7* [77]. *Bhagavad Gita* states that foods which aid longevity, knowledge, strength, health, happiness and love are preferred by people who are with a disposition of *Sattva* (goodness); and such foods would be full of juicy essences, made of fresh ghee or oil; thus highly nourishing to the mind and naturally tasteful—*Bhagavad Gita 17.8*. Foods that are very bitter, very sour, very salty, very hot, that cause burning sensation to the body, that dry up the body, that is burning hot; are the food items generally liked by those who are with disposition of *Rajas* (passion); and such foods cause mental stress, bodily suffering, pain and diseases—*Bhagavad Gita 17.9*. Foods that are cooked and kept for a long time, which have lost their natural taste, which have started to smell bad, which have become old, which is left over after consumption; are the foods liked by those with the disposition of *Tamas* (ignorance or inertia); and such foods also cause serious illnesses to the body—*Bhagavad Gita 17.10*. Further it’s also stated in *Bhagavad Gita* that consumption of food with the quality of goodness helps in cleansing the excess impurities of mind and thus make it become pure; whereas consumption of foods with the quality of passion arises an increased grief and consumption of foods with the quality of ignorance arises the levels of ignorance and inertia—*Bhagavad Gita 14.16* [77].

*Divyaprabandham,* the ancient Tamil text of sacred verses in praise of the Lord, states that the disposition of food is also changed by the disposition of the person who is either cooking it or is in contact with it [79]. The famous experiment carried out by Masuru Emoto on water and cooked rice, scientifically showed the same phenomenon, wherein the effect of an individual’s intention on water was tested through a double-blind test where a group of 2000 people focused positive intentions on water samples located inside an electromagnetic shielded room and similar water samples were set aside for controls. Observations adjudged by hundred neutral observers, clearly indicated that the ice crystals formed from the water exposed to positive intentions got higher scores for aesthetic appeal in comparison to the control water samples [80]. The *Anna Sukta*, literally meaning hymn to food, contained in the *Taittiriya Brahmana* and also in the *Rigveda samhita* states that the person who shares food obtains the same from the nature and person who does not share the food is devoured by the same food he partakes. It is also mentioned in *Anna Sukta* that the food forsakes a person who eats without giving and will always remain with a person who shares the food. Moreover, it is believed that the food given to the celestials, demigods and other human beings is set apart for the giver in this world and beyond [81]. Dietary regimen mentioned in ancient Indic texts considers food not just as physical nourishment but also as a means to realize the true function and purpose of life. As per *Chandogya Upanisad* (7–26-2) eating of pure form of food is important to lead a healthy life for which moderation in eating habits has been mentioned in *Yogatattva Upanisad*. While moderate eating is defined as *Yama* (control) and refraining from killing to eat is defined as *Niyama* (the rule). According to the *Bhagavad Gita*, over-eating is mentioned as a disqualifying habit for practicing yoga, which further transforms into an old saying of “one meal a day – man is a yogi (the balanced man), two meals a day – man is a *bhogi* (enjoyer) and three meals a day – man is a *rogi* (the sick man)” [82].

Sri Vedanta Desika’s *Ahara Niyama* is an elaborate discussion about food, majorly about its hygiene, and also gives a list of foods (fruits, vegetables, dried food) and waters that are forbidden from consumption ex. foods that over-stimulate (such as garlic, onions, radish, drumsticks, varieties of guards, greens, and mushrooms) cause an imbalance between the mind and body are forbidden from consumption. *Ahara Niyama* also gives a strict dietary code also including a list of constraints in the way food is being cooked and the machinery used for cooking. The texts of *Ahara Niyama* also have mentioned on “transformed foods” like *Appam* (puffed rice balls) and *Murukku* (a fried lentil pretzel): which are to be consumed in a short time after cooking preferably suitable during the pilgrimage. It is known that sweets like *Appam*/*Attirasam*, made out of jaggery, ground-nut, and ghee, are considered as wholesome and sattvic indigenous and ethnic South Indian foods [79]. The Materia Medica (*Padarthaguna Chintamani)* of Tamil Siddhars, compiled between the ninth and fourteenth centuries gives an extensive list of food-related terminologies, more specifically about the culinary practices like effects of cooked rice in combination with ghee, milk, curds, buttermilk, or tamarind. It also confirms garlic as a cure for skin diseases and phlegmatic conditions [83].

### Influence of Hindu traditional texts on South Indian ethnic foods

Based on the aforementioned explanations of the prominent Hindu traditional texts, it is very clear that food should be a means to primarily lead a healthy and holistic life. Also, according to the ancient Indic texts, food is a sacred entity and thus termed as “*Annam Parabrahma Swaroopam*” which translates to “food is the form of the greatest creator and symbolizes divine universal consciousness” and also “*Annam Bhootanama Jyeshtam tasmat annam sarvaushadam ucyate*” which translates to “food verily is the eldest born of beings and therefore it is the healing herb for all’ [84, 85]. Needless to say, food is thus given a very sacred position in ancient Hindu culture and its consumption had a hidden meaning, which later transformed into traditions. Since ethnic foods primarily consider the geographical availability of resources as a primary requirement and food preparations are supposed to be economically viable, invariably there would have been a traditional knowledge system behind their evolution. Traditional knowledge systems thus would have acted as guidelines and influenced the evolution of ethnic foods.

In the present work, we reviewed the classical Indic traditional text “*Ahara Niyama*” written by an ancient Indian theologian, poet and exponent of *vaisnavism* tradition, Sri Vedanta Desika (1268-1369), who proposed various food precepts, which in accordance to our knowledge are thought provoking and highly contemporary. Food precepts are in general, food related rules of partaking food and are generally intended to be followed while procuring, processing, preparing and consuming foods. The text primarily lists many forbidden foods and unhygienic culinary practices, with an intention to help people lead a life with spiritual discipline [79]. Sri Vedanta Desika is recorded to have been born in South India (Tamil Nadu), and also because of the fact that there is a lot of traditional similarity (especially amongst the Vegetarian Hindu community) across all the South Indian states, the present study is being designed to look at the ethnic foods of the geographical locations as shown in Fig. 1. Since ethnic foods are unique because of their deep rooted history basically constituted of traditional knowledge systems of South India, it is recommended that understanding the cultural roots would help in understanding food practices better, which would further help in understanding the population better.

Ethnographic studies done on the South Indian vegetarian foods in the present work, clearly indicates that the foods are perfect blend of all the tastes i.e., sweet, sour, salty, bitter and savoury. Moreover since the number of ingredients used to prepare a South Indian ethnic food is very high, food preparation process invariably needs to be a well calculated practice. All the aforementioned foods are also mentioned in Sri Vedanta Desika’s *Ahara Niyama* and other Indic traditional texts which explain about its age old lineage. Most of the foods are consumed even today across all the five states of South India, with some slight modifications of cooking methodologies. Though there appears an evident linguistic difference with reference to the names of ethnic foods, we found that there are negligible differences with reference to its culinary practices. Presently, few of the aforementioned food items are even being commercialized and sold in food outlets under the name of traditional authentic South Indian foods [86]. Though recipes for almost all the items are being there in wide publicity from many years, due to a gradual change in the mind set of the younger generations and also changing lifestyle demands, there is an increased preference towards readymade junk foods leading to an increased incidences of health upsets especially in the youth. Thus there is necessity for educating society about cultural roots and importance of ethnic foods. Moreover with the passing generations there is also a danger of losing the right culinary knowledge and present article is also an informative document in that direction. Similar such evidences are also being explained and drawn by earlier researches on different dimensions of traditional foods [87]. Earlier studies state that South Asian countries such as Bangladesh, India, Nepal, Pakistan and Sri Lanka possess a wide variety of ethnic dishes that are nutrient dense [88]. When analyzed and compared with Indian vegetarian ethnic food cultures, Srilanka also appears to possess rice, curry, cereal, grain and vegetable (fermented) based diets in the vegetarian foods. Additionally, traditional sweets made from rice, wheat and other cereals appear to be a similar feature to that of South Indian ethnic food tradition [89, 90]. Similarly, Pakistan is also observed to share common ethnic and traditional food practices with India and Afghanistan [88]. Fermented cereal based, dairy based, fruit/vegetable based foods are being reported as traditional ethnic foods of Pakistan [91]. Bangladeshis vegetarian ethnic foods also seem to be predominated by rice, wheat, green leafy vegetables, sweets, fruits, fermented beverages and pickles [92, 93]. Similarly, main vegetarian meal of Nepal also seems to have rice with pulses, vegetable curry, milk, curd and pickle. Additionally, as a part of traditional practice, fermented foods from split black gram/green gram, wheat/rice floor, soybean, fruits, vegetables and milk are also being reported in Nepal [94, 95]. Apart from South Asian countries, many other Asian countries are also reported to possess rich variety of ethnic food traditions. Cereal and milk based ethnic foods of Kyrgyzsthan [96] and over 100 varieties of ethnic rice based foods and desserts of Iran [97] seems to possess many similarities with South Asian ethnic foods especially in terms of ingredient usage and also appearance.

Religion is opined to account for 70% of human activities in South Asian Countries [98] and South Asia is reported to be having more vegetarian food choices because high percentages of Indians are pure vegetarians [99]. As opined by earlier researchers, food rules and laws (taboos) are influenced by religious beliefs/customs, which in turn are influenced by geographical location, environmental factors and availability of raw material for food preparation [100]. Having similarity of these factors would be a reason for most of the South Asian countries to have almost common ethnic food practices. However individual traditional beliefs across the countries would certainly influence minor variations of their ethnic foods. Similar to our observation in the present study, influence of an ancient text “Mahavamsa” on food practices of ethnic group of “Vedda” people of Srilanka [101] and reports of traditional Islamic texts being used as a knowledge resource to know that some vegetarian foods (dates, grapes, figs, pomegranate, cereal powder blend i.e., sattu, melons, carrots, pumpkins, lentils, powdered pulses and seeds i.e., qawoot and powdered sesame i.e., savigh and quince) are useful to treat male fertility related problems [102] strengthens our hypothesis of the influential role traditional knowledge systems play on the evolution of ethnic food cultures. However more in depth analysis would help understand similar such traditions probably associated with the ethnic food cultures of other South Asian countries.

## Conclusion

While food is certainly a person’s choice, it is known to be influenced by many factors viz., biological (hunger, appetite and taste), economical (cost, income and availability), physical (access, cooking skill and time), social (culture, tradition and meal pattern) and psychological (mood, stress and guilt) [103]. However, ethnic origin of a population seems to be a very strong influencing factor while looking at the phenomenon of food choices and there has been a steady rise in the research on food consumption traditions worldwide. Since culture is known to be a pivotal influential factor for lives, food consumption worldwide is tightly associated with cultures and traditions. Understanding and researching on ethnic foods is thus an important field of confluence and also need of hour. In further enhancing our understanding on ethnic foods and making them into a globally acceptable phenomenon, understanding their origin and the associated cultural backgrounds is highly warranted. Further, the developed knowledge has to be integrated with the scientific ideologies to make it more viable and acceptable. Though it is a general opinion that traditional practices are orthodox, not all of them can be rejected. In reality, traditional practices have been proven to be the best alternatives since they have formidable scientific relevance [104]. Moreover, in-depth understanding of traditional knowledge systems, undoubtedly would help life science researchers, in particular, because of the increasing demand for their research outputs in dealing with many health threat conditions, more evident from the current global pandemic—COVID-19 disease.

Ethnic foods are strongly influenced by the cultural setup of a particular region and the knowledge is generally transferred from generations, which subsequently becomes a tradition over time. In very early generations, since the general knowledge and know-how over several aspects were low, dependency on some form of traditional knowledge system seems to have been an invariant option. Food practices undoubtedly are thus a part of that knowledge flow, which obviously will have a deep rooted connection in culture.

Present work shows that traditional Indic texts has many food laws being laid and also constituted relevant food formulations, which perhaps would have been a necessity to suit to the then environmental conditions and people’s socio-economic status. However, since the traditions are carried from generations to generations, their unchanged existence even to date speaks about the traditional binding people evolve with. Though the present work only looks at one particular culture i.e., Hinduism, the aspect seems to have relevance in vast array of other cultures too. Moreover, the fact that there is a traditional continuation of differential usage of the food items across all the south Indian states, exactly as meant by Sri Vedanta Desika’s *Ahara Niyama,* is a very thought provoking observation. The order in which items are served and consumed follow a certain pattern, with optional exclusion of some items, which is predominantly decided by the occasion for which the items are prepared, psychological status of the person eating, prevailing climatic conditions and physiological status (assimilating capacity of digestive system). Thus, ethnic foods are understood to be prepared very carefully, so that they not only suffice the bodily nutritional demand but also can act as a natural medicament. Present theoretical review of the medicinal values all the food ingredients used in the preparation of South Indian vegetarian foods possess clearly proves this.

In our perspective, this study is a novel attempt clearly highlighting the importance of inculcating a different scientific acumen in understanding the domain of food. A thorough understanding of our theological and philosophical knowledge base would make the acumen transform into a holistic approach. Holistic approach of both science and philosophy would in turn help us revive our traditional ethnic food practices which ultimately help us in a better disease diagnosis—treatment regimens. This thought process confluence is need of the hour, given the uncertain health related threats present world is facing, where right kind of food certainly has an ability to bring right changes in one’s health.

### Limitation of the study and future scope

Present study is a micro-level understanding of only the vegetarian ethnic foods of South India, which is a limitation. However, the study sets a foundation on the strong influential role of religious traditions on the ethnic foods of a particular region. Similar such studies are possible even with non-vegetarian food practices across South India. The study opens up a window to further investigate and understand the Asian food cultural landscape more closely in an ethnographic perspective.

## Supplementary Information

**Additional file 1: Appendix 1**: Data procurement and analysis methodology employed for the ethnographic study presented in the manuscript. **Table S1**: Semi structured interviews conducted in-person at Temples on the traditional food preparations offered as Prasadam to the God. **Table S2**: Semi structured interviews and informal conversations conducted in-person or over telephone with individual Brahmin, Arya Vysya, Lingayath and Namboodiri families on their traditional food beliefs and practices.

**Additional file 2: Appendix 2**: Few photographs of Semi-structured interviews conducted at Temples (Photos taken only with the consent of the respondents)

## Data Availability

All the data generated during this study are included within the manuscript. Additional supporting materials are available in the Additional file 1: Appendix 1 and Additional file 2: Appendix 2. All the photographs are authors own contribution.
