# Supplementary material for: Vegetarian ethnic foods of South India: review on the influence of traditional knowledge
Source: J. Ethn. Food. 2022 Oct 21;9(1):42. doi: 10.1186/s42779-022-00156-1 (PMC9589551; doi:10.1186/s42779-022-00156-1)
Supplement: Supplementary file 1 — Additional file 1: Appendix 1: Data procurement and analysis methodology employed for the ethnographic study presented in the manuscript. Table S1: Semi structured interviews conducted in-person at Temples on the traditional food preparations offered as Prasadam to the God. Table S2: Semi structured interviews and informal conversations conducted in-person or over telephone with individual Brahmin, Arya Vysya, Lingayath and Namboodiri families on their traditional food beliefs and practices. [file 42779_2022_156_MOESM1_ESM.docx]

**Appendix - 1**

**Data procurement and analysis methodology employed for the ethnographic study presented in the manuscript**

**Table I: Semi structured interviews conducted in-person at Temples on the traditional food preparations offered as Prasadam to the God**

| **S.No** | **Temple / Institution** | **Tradition followed** | **Foods offered (*prasadam*) to god during** | | | | **Contact person, Age and Sex** | **Date of interaction** | **State** |
| --- | --- | --- | --- | --- | --- | --- | --- | --- | --- |
|  |  |  | **Morning** | **Afternoon** | **Evening** | **Night** |  |  |  |
| 1 | Govinda Raja Swamy Temple | Vaikhanasa Agama | Pongal | Neyyannam, Pulihora,  Paramannam | Aravani Guggillu Laddu | Dadhyodanam | Sri Varadarajan – 64/M | 07.11.21 | AP |
| 2 | Sri Raghavendra Swamy Mutt | Vaikhanasa Agama | Pongal,  Palam | Pulihora, Aravani Paramannam | Aravani Laddu | Dadhyodanam  Rasavali | Sri Sujayeendra – 55/M | 08.11.21 | AP |
| 3 | Sri Prasanna Anjaneya Swamy Temple | Vaikhanasa Agama | Pongal, Chitrannam | Neyyannam, Pulihora | Aravani Guggillu | Dadhyodanam | Sri Krishna Murthy Sandilya -58/M | 28.11.21 | TG |
| 4 | Sri Saraswati Temple | Shaivagama | Pongal | Neyyannam, Pulihora, Paramannam | Aravani Laddu | Dadhyodanam  Rasavali | Sri.Narayana Sharma – 60/M | 28.11.21 | TG |
| 5 | Sri Kodanda Rama Swamy Temple | Vaikhanasa Agama | Pongal | Neyyannam Pulihora Aravani Paramannam | Aravani Laddu | Dadhyodanam  Rasavali | Sri.Ramanujacharya – 54/M | 03.12.21 | KA |
| 6 | Karanji Anjaneya Temple | Vaikhanasa Agama | Idly, Milk | Kadambam, Sakkara Pongal, Puliogare, Dadhyodanam | Ten Palam,Usli | Dosa, Vada, | Srinivasa Bhattar – 40/M | 15.12.21 | KA |
| 7 | Bindiganavile  Channakeshava Temple | Pancharatra Agama | Pongal | Kadambam, Sakkara Pongal, Puliogare, Dadhyodanam, Rice and Ghee, Mor Kozambu, Rasam, Sambar | Fruits | Rasam, Rice with Ghee | Srivathsa Bhattar – 48/M, Balaji Bhattar – 66/M | 20.12.21 | KA |
| 8 | Kalyana Srinivasa Temple | Pancharatra Agama | Pongal | Kadambam, Sakkara Pongal, Puliogare, Dadhyodanam | Ten Palam | Dosa, Vada | Balaji Bhattar – 68/M | 27.12.21 | KA |
| 9 | Pattabhirama Temple | Pancharatra Agama | Pongal | Kadambam, Sakkara Pongal, Puliogare, Dadhyodanam | Fruits | Avalakki, Usli, Lemon Rice | Mr.Srinivasan – 64/M | 08.01.22 | TN |
| 10 | Guruvayoor Krishna Temple | Rahasya Vishnu Tantra | Plantain and Sugar, Usha Payasa Neivedyam | Rice, Morkozambu, Palpayasam, Sarkarapayasam | Neipayasam, Butter | Trimaduram Appam , Ada, Avil | Mr. Gopalakrishnan – 60/M | 15.01.22 | KL |
| 11 | Sri Venkateshwara Temple | Vaikhanasa Agama | Ten Palam, Idly, Pongal | Kadambam, Sakkara Pongal, Puliogare, Dadhyodanam, Rice and Ghee, Rasam, Sambar | Usli  Fruits | Dosa, Lemon Rice | Sri Srinivasacharyalu – 46/M | 22.01.22 | AP |
| 12 | Srirangam | Pancharatra Agamam | Sambar,Dosa, Chatni | Milagu Rasam, Koottu, Rice, Papad, Athirasam |  | Aravani, Sakkarapongal | Sri Sampath Bhattar – 65/M | 29.01.22 | TN |
| 13 | Parthasarathi Temple | Vaikhanasa Agamam | Raw Milk, Pongal, Rice, Puliyogarai, Dadhyodanam, Sakkarapongal | Rice, Kuzambu, Rasam, Curd | Idli, Dosa, Sundal Vada | Aravani, Sakkarapongal | M.A.Venkatakrishnan – 66/M & Ramanujam – 60/M | 30.01.22 | TN |
| 14 | Varadaraja Temple | Pancharatra Agama | Idli, Vada, Dosa | Puliyogarai, Sakkarapongal, Rice, Kozambu, Rasam | Murukku, Tenkuzal | Aravani | U.Ve. Ananthapadmanabhachariar – 64/M | 05.02.22 | TN |
| 15 | Gopalkrishnan Temple | Vishnu Tantra | Payasa Neivedyam | Rice, Morkozambu, Palpayasam, Sarkarapayasam | Neipayasam, Butter | Appam , Ada, Avil | Sri.Namboodri – 60/M | 06.02.22 | KL |
| 16 | Kanyakaparameshwari Temple | Vaikhanasa Agamam | Pongal, Vada | Neyyannam Pulihora, Koora, Appadam | Aravani,  Laddu, Kova | Perugu Vada, Rasavali | Sri.Pradeep – 58/M | 18.02.22 | KA |
| 17 | Sri Shivalayam Temple | Vaikhanasa Agamam | Pongal | Kadambam, Rice and Ghee, Mor Kozambu, Rasam, Sambar | Fruits with milk | Rasam, Rice with Ghee | Sri.Umashankar – 40/M | 22.02.22 | KA |
| 18 | Sri Subrahmanyaswamy Temple | Vaikhanasa Agama | Pongal | Pulihora, Paramannam, | Laddu | Dadhyodanam | Sri.Skanda Murthy – 50/M | 23.02.22 | KA |
| 19 | Sri Gopalalrishna Temple | Vaikhanasa Agama | Pongal | Neyyannam Pulihora Aravani Paramannam, | Aravani Laddu | Dadhyodanam  Rasavali | Sri.Narayanan Theertha – 65/M | 25.02.22 | KL |
| 20 | Sri Kannur Ganesha Temple | Vaikhanasa Agama | Milk | Pongal, Puliogare, Dadhyodanam | Usli | Milk and Palam | Naryan Nambudiri – 50/M | 27.02.22 | KL |

**Table II: Semi structured interviews and informal conversations conducted in-person or over telephone with individual Brahmin, Arya Vysya, Lingayath and Namboodiri families on their traditional food beliefs and practices.**

| **S.No** | **Ethnic group, tradition followed** | **Foods prepared at home during**  **(* if the food is offered to God before consumption)** | | | | **Contact person, Age and Sex** | **Mode & Period of interaction** | **State** |
| --- | --- | --- | --- | --- | --- | --- | --- | --- |
|  |  | **Breakfast** | **Lunch** | **Evening snacks** | **Dinner** |  |  |  |
| 1 | Brahmins, Vaishnava | Pongal*, Idly, Vadai*,Tayir Vadai | Nei Sadam*, Adirasam (only prepared during ancestral ceremonies), Aravani*, Aviyal*, Sidai*, Kulumbu, Kariyamudu,Oorugaai Parappuvial*, Satramudu, Appadam | Tirukannamudu*, Tirattupal*, Varuval, Vadam and Vatral | Sadam with Sambar and Rasam,  Dadhyodanam* | Mrs.Soundara Rajan, 66/F | Telephonic conversation  19.10.20 | TN |
| 2 | Brahmins, Smartha | Dosa, Idly, Upma, Puffed rice, Pongal*, Vada*, Rasavali* | Pulihora*, Paramannam*, Chitrannam*Vadiyam, Majjiiga, Pulusu, Gojju, Vepudu, Koora, Sambar, Uragaya, Rasam, Appadam, Ariselu*(ancestral ceremonies), Purnalu* *(ancestral ceremonies) | Undalu*,  Guggillu*, Laddu*, | Annam with Pappu and Rasam | Mrs.Jayalakshmi, 72/F & Sri.Srinivasa Murthy, 73/M | In-person  22.10.20 | AP |
| 3 | Brahmins, Niyogi | Pongal*, Vada*, Dosa, Idly | Pulihora*, Vadiyam, Majjiiga, Pulusu, Gojju, Vepudu, Koora, Sambar, Uragaya, Rasam | Kajjaya, (ancestral ceremonies)  Guggillu* Laddu* Kova* | Annam with Sambar and Rasam, Dadhyodanam* | Mrs.Prasanna Lakshmi, 66/F | In-person  18.11.20 | TG |
| 4 | Brahmins, Madhvas | Idly, Pongal*, Vada*, Dosa | Pulihora*, Sandigae*, Majjiiga, Huli, Gojju, Palya, Sambar, Rasam | Kajjaya*,  Sundal*, Laddu* | Anna with Sambar and Rasam, Dadhyodanam* | Prof.Madhwaraj, 55/M | In-person  22.11.20 | KA |
| 5 | Brahmins, Badaganadu | Dosa, Idly, Pongal* Vada*, Upma, | Usili, Pulihora*, Sandigae, Gojju, Bele Palya, Sambar, Rasam, Dadhyodanam* | Rasavali* | Anna with Sambar and Rasam | Dr. Aravinda, 56/M | In-person  29.11.20 | KA |
| 6 | Lingayatha, Shivagama | Dosa, Idly, Vada*Pongal* | Usili, Pulihora*, Palya, Sambar, Rasam | Sandigae*, Sundal* | Perugu Vada,  Annam with Pappu, Rasam | Mr. Prasanna Kumar, 50/M | Telephonic conversation  29.11.20 | KA |
| 7 | Brahmin, Nambudri | Idly, Puttu, Pongal* Vada* | Neichoorru*,Aviyal*, Varavu, Poricha*, Satramudu | Atirasam*  Unni Appam* Laddu* | Pal Payasam*  Dadhyodanam* | Mrs.Asha Nambudripad, 48/F | Telephonic conversation  10.12.20 | KL |
| 8 | Brahmin, Vaishnava | Pongal*,Vadai*, Dosa, Tayir Vadai | Neyyannam*, Pulihora*, Vadiyam, Sambar, Uragaya, Rasam | Adirasam *  Guggillu* Laddu* Kova* | Dadhyodanam* Sadam with Satramudu, Rasavali | Mrs.Chandralekh, 50/F | In-person  18.12.20 | TN |
| 9 | Brahmins, Badaganadu | Idly, Vada, Dosa,Payasam* | Neyyannam*, Pulihora*, Aravani, Paramannam*, Vadiyam, Majjiiga, Pulusu, Gojju, Vepudu, Koora, Sambar, Uragaya, Rasam, Appadam | Aravani, Adirasam (only prepared during ancestral ceremonies)  Guggillu* Laddu* Kova* | Dadhyodanam* Tayir Vada, Rasavali | Mr. Srinivasa Rao, 55/M | In-person  22.12.20 | AP |
| 10 | Arya Vysya | Dosa, Idly, Pongal*, Vada*, Rice Savigae, Avalakki | Pulihora*, Chitrannam*,Aravani, Paramannam*, Vadiyam, Majjiiga, Pulusu, Gojju, Vepudu, Koora, Sambar, Uragaya, Rasam, Appadam | Aravani, Ariselu, Undalu*, Puffed Rice,  Guggillu*, Laddu*, Kova* | Dadhyodanam*, Perugu Vada, Rasavali | Ms. Dhanalakshmi, 48/F | In-person  24.12.20 | KA |
| 11 | Lingayatha, Shivagama | Pongal*, Vada* | Neyyannam*, Pulihora*, Vadiyam, Majjiiga, Pulusu, Gojju, Vepudu, Koora, Sambar, Uragaya, Rasam | Adirasam (ancestral ceremonies)  Guggillu* Laddu* Kova* | Dadhyodanam* Perugu Vada, Rasavali | Mr.Manjunatha, 55/M | In-person  26.12.20 | KA |
| 12 | Brahmin, Nambudri | Idly, Puttu, Pongal* Vada* | Aviyal*, Varavu, Poricha* | Atirasam* (ancestral ceremonies)  Unni Appam* Laddu* | Pal Payasam*  Dadhyodanam* | Mr.Arun, 66/M | Telephonic conversation  03.01.21 | KL |
| 13 | Brahmin, Vaishnava | Dosa, Idly, Pongal* Vada* | Nei Sadam*, Adirasam (only prepared during ancestral ceremonies), Aravani*, Aviyal*, Sidai*, Kulumbu, Kariyamudu,Oorugaai | Tirukannamadai*, Sukhiyan | Sadam with Satramudu, Dadhyodanam* | Mrs.Narayani, 68/F | In-person  12.01.21 | TN |
| 14 | Brahmins, Vaishnava | Dosa, Idly, Pongal* | Usili, Pulihora*, Vadiyam, Pulusu,  Sambar, Rasam | Vadiyalu, Palam | Perugu Vada,  Annam with Pappu | Mrs. Kamakshi, 62/F | In-person  15.01.21 | KA |
| 15 | Brahmins, Smartha | Dosa, Idly, Pongal* Vada* | Usili, Pulihora*, Vadiyam, Majjiiga, Pulusu, Gojju, Vepudu, Sambar, Rasam | Guggillu*  Vadiyalu | Perugu Vada,  Palam with honey | Mr.Nagaraja Shastry, 70/M | In-person  19.01.21 | KA |
| 16 | Brahmins, Niyogi | Pongal*, Vada*,Idly, Dosa | Neyyannam, Chitrannam*, Pulihora*, Vadiyam, Sambar, Uragaya, Rasam | Atirasam,  Guggillu* Laddu* Kova* | Dadhyodanam* Perugu Vada, Rasavali | Mr.Madhukar, 52/M | In-person  29.01.21 | TG |
| 17 | Brahmins, Badaganadu | Dosa, Idly, Pongal*, Vada*. | Pulihora*, Aravani, Paramannam, Chitrannam*, Vadiyam, Majjiiga, Pulusu, Gojju, Vepudu, Koora, Sambar, Uragaya, Rasam, Appadam | Aravani, Ariselu, Undalu*,  Guggillu*, Laddu*, Kova* | Dadhyodanam*, Perugu Vada, Rasavali | Mrs.Sumithra, 55/F | In-person  30.01.21 | AP |
| 18 | Arya Vysya | Pongal* , Vada*, Dosa, Upma, Puffed Rice | Neyyannam, Pulihora*, Vadiyam, Majjiiga, Pulusu, Gojju, Vepudu, Koora, Sambar, Uragaya, Rasam | Adirasam (ancestral ceremonies)  Guggillu* Laddu* Kova* | Dadhyodanam* Perugu Vada, Rasavali | Mr.Pradeep Shetty, 50/M | In-person  13.02.21 | KA |
| 19 | Lingayatha, Shivagama | Idly, Pongal* Vada* | Pulihora*, Vadiyam, Majjiiga, Pulusu, Gojju, Vepudu, Sambar, Rasam | Kajjaya,  Guggillu* Laddu* | Dadhyodanam* Perugu Vada, | Ms.Gangamma, 55/F | In-person  16.02.21 | KA |
| 20 | Brahmin, Nambudri | Idly, Dosa, Pongal* Vada* | Neichorru,Aviyal*, Varavu, Poricha* | Atirasam* (ancestral ceremonies)  Unni Appam* Laddu* | Nei Payasam*,  Dadhyodanam* | Mrs.Janaki, 68/F | Telephonic  20.02.21 | KL |
| 21 | Brahmin, Vaishnava | Dosa, Idly, Pongal*, Vada*. | Pulihora*, Paramannam, Vadiyam, Majjiiga, Pulusu, Gojju, Bele Palya, Sambar, Uppinakay, Rasam, Appadam | Undalu*,  Guggillu*, Laddu*, Kova* | Dadhyodanam*, Rasavali | Ms.Gayathri Mukundam, 54/F | Telephonic  22.02.21 | KA |
| 22 | Brahmins, Badaganadu | Pongal* Vada* | Neyyannam, Pulihora*, Appala, Gojju, Palya, Sambar, Uppinakay, Rasam | Adirasam (ancestral ceremonies)  Sundal, Laddu* Kova* | Dadhyodanam* Perugu Vada, Rasavali | Mr.Sesha Sastry, 60/M | In-person  26.02.21 | KA |
| 23 | Brahmins, Vaishnava | Pongal* Vada* | Neisadam, Pulihora*, Kuzhambu, Paruppusili | Adirasam (only prepared during ancestral ceremonies)  Laddu* | Dadhyodanam* Tayir Vada, Rasavali | Dr.Shyamsunder, 65/M | Telephonic  02.03.21 | TN |
| 24 | Brahmins, Smartha | Dosa, Idly, Pongal*, Vada*. | Pulihora*, Aravani, Paramannam, Gojju, Sambar, Uppinakay, Rasam, Appala | Ariselu, Undalu*,  Laddu*, Kova* | Dadhyodanam*, Perugu Vada, Rasavali | Prof.Meenakshi Sundaram, 50/M | In-person  04.03.21 | KA |
| 25 | Brahmins, Niyogi | Pongal* Vada* | Neyyannam, Pulihora*, Vadiyam, Majjiiga, Pulusu, Gojju, Vepudu, Koora, Sambar, Uragaya, Rasam | Atirasalu,  Guggillu* Laddu* Kova* | Dadhyodanam* Perugu Vada, Rasavali | Mrs.Praneetha, 48/F | In-person  12.03.21 | TG |
| 26 | Brahmins, Havyak | Pongal* Vada*, Dosa, Idly, Pathurodae, kadabu* | Thuppada Anna, Pulihora*,Huli, Thambali,  Gojju, Rasam | Payasam*, Sabbakki, Kesari bath*, Sundal, Laddu* Kova* | Anna with Thavve and Rasam | Dr.Sridhar Khurse, 58/M | In-person  15.03.21 | KA |
| 27 | Arya Vysya | Idly, Pongal*, Vada*, Dosa, Avalakki | Chitrannam*, Pulihora*, Vadiyam, Majjiiga, Pulusu, Gojju, Vepudu, Sambar, Rasam | Kajjaya (ancestral ceremonies)  Guggillu* Laddu* | Dadhyodanam* Perugu Vada, | Mrs.Rekha Gayathri, 63/F | In-person  19.03.21 | AP |
| 28 | Lingayatha, Shivagama | Pongal* Vada* | Neyyannam, Pulihora*, Appala, Majjiigae Huli, Gojju, Sambar, Uppinakay, Rasam | Adirasam (ancestral ceremonies)  Sundal, Laddu* Kova* | Dadhyodanam* Perugu Vada, Rasavali | Mrs.Uma Bharathi, 63/F | In-person  12.04.21 | KA |
| 29 | Brahmin, Nair | Idly, Dosa, Pongal*, Vada*, Appam*, Puttu | Sadam, Theil, Neichorru,Aviyal*, Varavu, Poricha* | Atirasam*  Unni Appam* Laddu* | Sadam with Pachadi, Payar Kanji,  Dadhyodanam* | Prof.Sreeja, 52/F | In-person  18.05.21 | KL |
| 30 | Arya Vysya | Idly, Pongal* Vada* | Neyyannam, Pulihora*, Vadiyam, Majjiiga, Pulusu, Gojju, Vepudu, Koora, Sambar, Uragaya, Rasam | Kajjaya,  Guggillu* Laddu* Kova* | Dadhyodanam* Perugu Vada, Rasavali | Mrs.Kanyaka, 55/F | In-person  22.06.21 | AP |
| 31 | Lingayatha, Shivagama | Pongal*, Vada*,Usili, Idly | Neyyannam, Pulihora*, Vadiyam, Gojju, Vepudu, Palya, Sambar, Uragaya, Rasam | Atirasam (ancestral ceremonies)  Guggillu* Laddu* Kova* | Dadhyodanam* Perugu Vada, Rasavali | Mrs. Gowri, 56/F | In-person  23.06.22 | KA |
| 32 | Arya Vysya | Idly, Pongal* Vada* | Chitrannam*, Pulihora*, Aravani, Paramannam, Vadiyam, Majjiiga, Pulusu, Gojju, Vepudu, Koora, Sambar, Uragaya, Rasam, Appadam | Aravani, Ariselu, Undalu*,  Guggillu*, Laddu*, Kova* | Dadhyodanam*, Perugu Vada, Rasavali | Mrs.Sundaramma, 70/F | In-person  04.07.21 | TG |
| 33 | Lingayatha, Shivagama | Pongal*, Vada*, Idly, Dosa | Neyyannam, Pulihora*, Majjiigae Huli, Gojju, Koora, Sambar, Uragaya, Rasam | Adirasam (ancestral ceremonies)  Guggillu* Laddu* Kova* | Dadhyodanam* Perugu Vada, Rasavali | Mr.Mahalingam, 65/M | In-person  15.07.21 | KA |
| 34 | Brahmins, Smartha | Idly, Pongal* Vada*, Upma, Dosa | Pulihora*,Anna,  Gojju, Vepudu, Sambar, Rasam | Kajjaya (ancestral ceremonies)  Guggillu* Laddu* | Dadhyodanam* Anna with Rasam, Rasavali* | Smt.Geeta Nagaraj, 50/F | In-person  28.07.21 | KA |
| 35 | Brahmins, Vaishnava | Pongal*, Vada*,Idali, Tayir Vada | Neyyannam, Pulihora*, Aravani, Paramannam, Pulikariamudu, Pulippakoottu, Uroogayi, Rasam, Appadam | Aravani, Adirasam, Varuval | Sadam with Sambar and Rasam,  Dadhyodanam* | Mrs.Jayanthi, 58/F | Telephonic conversation  19.10.20 | TN |
| 36 | Brahmins, Smartha | Dosa, Idly, Pongal*, Vada*. | Pulihora*, Aravani, Paramannam, Anna with Sambar, Uppinakaya, Rasam, Appadam | Aravani, Ariselu, Undalu*,  Guggillu*, Laddu*, Kova* | Dadhyodanam*, Perugu Vada, Rasavali | Dr.Sainath, 55/M | In-person  22.10.20 | KA |
| 37 | Brahmins, Niyogi | Pongal*, Vada*, Perugu Vada, Idly, Dosa | Neyyannam, Chitrannam* Pulihora*, Vadiyam, Majjiiga, Pulusu, Gojju, Vepudu, Koora, Sambar, Uragaya, Rasam | Adirasam (ancestral ceremonies)  Guggillu* Laddu* Kova* | Perugu Vada, Rasavali* | Mrs.Nirmala, 68/F | In-person  18.11.20 | TG |
| 38 | Brahmins, Badaganadu | Idly, Pongal* Vada* | Chitrannam*, Pulihora*, Anna Huli, Gojju, Palya Sambar, Rasam | Kajjaya (ancestral ceremonies)  Sundal Laddu* | Dadhyodanam*, Anna with Rasam | Prof.Vishwanath, 50/M | In-person  22.11.20 | KA |
| 39 | Arya Vysya | Dosa, Idly, Pongal* Vada* | Usili, Pulihora*, Huli, Gojju, Palya, Sambar, Rasam | Rasavali, Sandigae | Annam with Pappu | Mr. Sitaram Shetty, 52/M | Telephonic conversation  29.11.20 | KA |
| 40 | Lingayatha, Shivagama | Dosa, Idly, Pongal* | Usili, Pulihora*, Anna   Sambar, Rasam | Vadiyalu, Palam | Perugu Vada,  Annam with Pappu | Mr.Shiva Kumar, 61/M | Telephonic conversation  29.11.20 | KA |
| 41 | Brahmin, Nambudri | Idly, Dosa, Pongal* Vada*, Appam* | Neichorru, Aviyal*, Varavu, Poricha* | Atirasam* (ancestral ceremonies)  Unni Appam* Laddu* | Nei Payasam*,  Dadhyodanam* Rasavali | Prof.Surendra, 58/M | In-person  10.12.20 | KL |
| 42 | Brahmin, Vaishnava | Pongal*, Vada*, Idali, Dosa | Neyyannam, Pulihora*, Pulippukoottu, Parappu Usili, Sambar | Adirasam (ancestral ceremonies)  Guggillu* Laddu* Kova* | Dadhyodanam* Sadam with Satramudu, Rasavali* | Mrs.Parthasarathi Narayanan, 63/F | In-person  18.12.20 | TN |
| 43 | Brahmins, Badaganadu | Pongal* Vada*, Idly, dosa, Avalakki | Neyyannam, Chitrannam*, Pulihora*, Aravani, Paramannam, Vadiyam, Majjiiga, Pulusu, Gojju, Vepudu, Koora, Sambar, Uragaya, Rasam, Appadam | Aravani, Adirasam (only prepared during ancestral ceremonies)  Guggillu* Laddu* Kova* | Dadhyodanam* Tayir Vada, Rasavali | Mr. Lakshmana Murthy, 70/M | In-person  22.12.20 | AP |
| 44 | Arya Vysya | Dosa, Idly, Pongal*, Vada*. | Chitrannam*, Pulihora*, Aravani, Paramannam, Anna with Sambar, Uppinakaya, Rasam, Appadam | Aravani, Ariselu, Undalu*,  Sundal, Laddu*, Kova* | Dadhyodanam*, Rice with Rasam, Rasavali | Mrs. Gajalakshmi, 62/F | In-person  24.12.20 | KA |
| 45 | Lingayatha, Shivagama | Pongal*, Vada*, Idly, Dosa | Tuppadanna, Chitranna*, Pulihora*, Anna Sambar, Sandigae, Rasam | Adirasam (ancestral ceremonies)  Guggillu* Laddu* Kova* | Dadhyodanam* Perugu Vada, Rasavali | Mr.Rajesh, 51/M | In-person  26.12.20 | KA |
| 46 | Brahmin, Nambudri | Idly, Dosa, Pongal* Vadai*, Puttu, Appam* | Neichorru, Aviyal*, Varavu, Poricha*, Pulikariamudu, Parappu Usili | Atirasam* (ancestral ceremonies)  Unni Appam* Laddu* | Dadhyodanam*, Sadam with Satramudu | Mr. Krishna, 54/M | Telephonic conversation  03.01.21 | KL |
| 47 | Brahmin, Vaishnava | Dosa, Idali, Pongal* Vadai*, Dadhyodanam* | Sadam, Pulippakoottu, Poritta koottu, Avial, Kolumbu, Satramudu, Parappu Usili | Tirukannamuudu*, Varuval | Sadam with Satramudu, | Mrs.Narayani, 70/F | In-person  12.01.21 | TN |
| 48 | Brahmins, Vaishnava | Dosa, Idly, Pongal*, Perugu Vada* | Usili, Pulihora*, Anna with Sambar, Rasam, | Sandigae, Palam* | Anna with Pappu, Rasam | Mrs. Kamakshi, 61/F | In-person  15.01.21 | KA |
| 49 | Brahmins, Smartha | Dosa, Idly, Pongal*, Vada*, Payasam* | Usili, Pulihora*, Vadiyam, Majjiiga, Pulusu, Gojju, Vepudu, Sambar, Rasam | Sundal, Rasavali | Anna with Rasam,  Palam with honey | Mr.Nagabhushan, 49/M | In-person  19.01.21 | KA |
| 50 | Brahmins, Niyogi | Pongal*, Vada*, Upma, Idly, Dosa | Neyyannam, Pulihora*, Vadiyam, Sambar, Uragaya, Rasam | Adirasam (ancestral ceremonies)  Guggillu* Laddu* Kova* | Dadhyodanam* Perugu Vada, Annam with Sambar, Rasam, Rasavali | Mrs.Manjula Vani, 50/F | Telephonic conversation  29.01.21 | TG |
| 51 | Brahmins, Badaganadu | Dosa, Idly, Pongal*, Vada*, Upma | Pulihora*, Chitrannam*, Aravani, Paramannam, Vadiyam, Majjiiga, Pulusu, Gojju, Vepudu, Koora, Sambar, Uragaya, Rasam, Appadam | Aravani, Ariselu, Undalu*,  Guggillu*, Laddu*, Kova* | Dadhyodanam*, Perugu Vada, Rasavali | Mrs.Sumithra, 52/F | In-person  30.01.21 | AP |
| 52 | Arya Vysya | Pongal*, Vada*,Payasam*, Idly, Vada, Avalakki | Neyyannam, Chitrannam*, Pulihora*, Vadiyam, Majjiiga, Pulusu, Gojju, Vepudu, Koora, Sambar, Uragaya, Rasam | Adirasam (ancestral ceremonies)  Guggillu* Laddu* Kova* | Dadhyodanam* Perugu Vada, Annam with pulusu, Rasam, Rasavali | Mr.Yogendra, 48/M | In-person  13.02.21 | AP |
| 53 | Lingayatha, Shivagama | Idly, Pongal* Vada* | Pulihora*, Anna with Sambar, Gojju, Palya, Rasam | Kajjaya (ancestral ceremonies)  Sundal* Laddu* | Dadhyodanam* Rice with Rasam | Mrs. Gangavathi, 55/F | In-person  16.02.21 | KA |
| 54 | Brahmin, Nambudri | Idly, Dosa, Pongal*, Vada*, Puttu, Appam* | Neichorru, Aviyal*, Varavu, Poricha*, Avial | Tirukannamudu, Thaen Palankal,  Unni Appam* Laddu* | PalPayasam*,  Dadhyodanam* | Mrs.Parvathamma, 85/F | Telephonic  20.02.21 | KL |
| 55 | Brahmin, Vaishnava | Dosa, Idly, Pongal*, Vada*. | Pulihora*, Aravani, Paramannam, Vadiyam, Majjiiga, Huli, Gojju, Palya, Sambar, Rasam, Appala | Aravani, Ariselu, Undalu*,  Sundal*, Laddu*, Kova* | Dadhyodanam*, Rice with Rasam, Usili, Rasavali | Mrs.Leelavathi, 66/F | Telephonic  22.02.21 | KA |
| 56 | Brahmins, Badaganadu | Pongal*, Vada*, Paramanna*, Idly, Dosa | Pulihora*, Chitrannam*, Anna with Sambar, Gojju, Palya, Rasam, Dadhyodanam* | Adirasam (ancestral ceremonies)  Laddu* Kova* | Dadhyodanam* Rice with Rasam | Mr.Karthik Rao, 51/M | In-person  26.02.21 | KA |
| 57 | Brahmins, Vaishnava | Pongal*,Vada*, Idali, Tayir Vadai, Dosa, Sugiyan* | Sadam, Pulippakoottu, Poritta koottu, Avial, Kolumbu, Satramudu, Parappu Usili | Aravani, Adirasam , Tirukannamudu,  Laddu* Kova* | Dadhyodanam* Tayir Vada, Rasavali | Mr.Karunakaran, 55/M | Telephonic  02.03.21 | TN |
| 58 | Brahmins, Smartha | Dosa, Idly, Pongal*, Vada*. | Pulihora*, Aravani, Paramannam, Vadiyam, Majjiiga, Huli, Gojju, Palya, Sambar, Rasam, Appala | Aravani, Ariselu, Sundal, Rasavali | Dadhyodanam*, Perugu Vada, Rasavali | Mr. Prabhakara Rao, 68/M | In-person  04.03.21 | KA |
| 59 | Brahmins, Niyogi | Pongal*, Vada*,Idly, Dosa, Upma | Neyyannam, Pulihora*, Vadiyam, Majjiiga, Pulusu, Gojju, Vepudu, Koora, Sambar, Uragaya, Rasam | Adirasam, Purnalu (ancestral ceremonies)  Guggillu* Laddu* Kova* | Dadhyodanam* Perugu Vada, Rasavali | Mrs.Sri Lakshmi, 52/F | In-person  12.03.21 | TG |
| 60 | Brahmins, Badaganadu | Pongal*, Vada*, Upma, Idly, Dosa, upma | Tuppadanna, Pulihora*, Anna Sambar, Sandigae, Rasam | Adirasam (ancestral ceremonies)  Guggillu* Laddu* Kova* | Dadhyodanam* Perugu Vada, Rasavali | Mr. Raghunatha Rao, 75/M | Telephonic  15.03.21 | KA |
| 61 | Arya Vysya | Idly, Pongal*, Vada*, Dosa | Pulihora*, Chitrannam*, Vadiyam, Majjiiga, Pulusu, Gojju, Vepudu, Sambar, Rasam | Kajjaya (ancestral ceremonies)  Guggillu* Laddu* | Dadhyodanam* Perugu Vada, | Mrs.Rekha Kumari, 49/F | In-person  19.03.21 | AP |
| 62 | Lingayatha, Shivagama | Pongal* Vada* | Pulihora*, Aravani, Paramannam, Anna, Majjiigae Huli, Gojju, Palya, Sambar, Rasam, Appala | Adirasam (ancestral ceremonies)  Sundal* Laddu* Kova* | Dadhyodanam* Perugu Vada, Rasavali | Mrs.Sailaja, 58/F | In-person  12.04.21 | KA |
| 63 | Brahmin, Nambudri | Idly, Dosa, Pongal*, Vada*, Thaen Palankal | Neichorru, Aviyal*, Varavu, Poricha*, Avial | Atirasam* ,  Unni Appam*,Tirattupal*, Laddu* | Nei Payasam*,  Dadhyodanam* | Mr. Nagendra, 59/M | In-person  18.05.21 | KL |
| 64 | Arya Vysya | Idly, Pongal* Vada*, Dosa, Upma | Neyyannam, Chitrannam*, Pulihora*, Vadiyam, Majjiiga, Pulusu, Gojju, Vepudu, Koora, Sambar, Uragaya, Rasam | Adirasam (ancestral ceremonies) ,  Guggillu*, Laddu*, Kova* | Dadhyodanam* Perugu Vada, Rasavali | Mrs.Kathyayini, 56/F | In-person  22.06.21 | AP |
| 65 | Lingayatha, Shivagama | Pongal*, Vada*, Idly, Dosa | Rice, Pulihora*, Vadiyam, Sambar, Pappu, Rasam | Adirasam (ancestral ceremonies)  Sundal*, Laddu* Kova* | Dadhyodanam* Perugu Vada, Rasavali | Mrs. Mohana Sruthi, 56/F | In-person  23.06.22 | KA |
| 66 | Arya Vysya | Idly, Pongal* Vada*, Avalakki | Pulihora*, Aravani, Paramannam, Vadiyam, Majjiiga, Pulusu, Gojju, Vepudu, Koora, Sambar, Uragaya, Rasam, Appadam | Aravani, Ariselu, Undalu*,  Guggillu*, Laddu*, Kova* | Dadhyodanam*, Perugu Vada, Rasavali, Rice Rasam | Mr.Pavan Shetty, 55/F | Telephonic  04.07.21 | TG |
| 67 | Lingayatha, Shivagama | Pongal* Vada* | Tuppadanna, Pulihora*, Anna Sambar, Sandigae, Rasam | Adirasam (ancestral ceremonies)  Sundal* Laddu* Kova* | Dadhyodanam* Perugu Vada, Rasavali | Mr.Sivaraj, 54/F | In-person  15.07.21 | KA |
| 68 | Brahmins, Niyogi | Idly, Pongal* Vada* | Pulihora*, Vadiyam, Majjiiga, Pulusu, Gojju, Vepudu, Sambar, Rasam | Kajjaya (ancestral ceremonies)  Guggillu* Laddu* | Dadhyodanam* Perugu Vada, | Dr.Pramod Joshi,60/M | In-person  28.07.21 | TG |
| 69 | Brahmins, Vaishnava | Pongal*, Vada*,Idali, Tayir Vada | Neyyannam, Pulihora*, Aravani, Paramannam, Pulikariamudu, Pulippakoottu, Uroogayi, Rasam, Appadam | Aravani, Adirasam, Varuval | Sadam with Sambar and Rasam,  Dadhyodanam* | Mrs.Vasantha, 66/F | Telephonic conversation  19.10.20 | TN |
| 70 | Brahmins, Smartha | Dosa, Idly, Pongal*, Vada*. | Pulihora*, Aravani, Paramannam, Vadiyam, Majjiiga, Pulusu, Gojju, Vepudu, Koora, Sambar, Uragaya, Rasam, Appadam | Aravani, Ariselu, Undalu*, Purnalu(ancestral ceremonies),  Guggillu*, Laddu*, Kova* | Dadhyodanam*, Perugu Vada, Rasavali | Mrs.Vijayalakshmi, 63/F | In-person  22.10.20 | AP |
| 71 | Brahmins, Niyogi | Pongal* Vada* | Neyyannam, Pulihora*, Vadiyam, Majjiiga, Pulusu, Gojju, Vepudu, Koora, Sambar, Uragaya, Rasam | Adirasam (ancestral ceremonies)  Guggillu* Laddu* Kova* | Perugu Vada, Rasavali* | Mrs.Krishna Kumari,67/F | In-person  18.11.20 | TG |
| 72 | Brahmins, Badaganadu | Idly, Pongal*, Vada*, Dosa | Pulihora*, Vadiyam, Gojju, Palya, Sambar, Rasam | Kajjaya (ancestral ceremonies), Rasavali, | Dadhyodanam* Perugu Vada, Rice with Rasam | Mr. Parashurama, 58/M | In-person  22.11.20 | KA |
| 73 | Arya Vysya | Dosa, Idly, Pongal* Vada* | Usili, Pulihora*, Majjiigae Huli , Gojju, Palya, Sambar, Rasam | Sundal, Rasavali | Perugu Vada,  Annam with Sambar | Mr. Ramakanth Shetty, 69/M | Telephonic conversation  29.11.20 | KA |
| 74 | Lingayatha, Shivagama | Dosa, Idly, Pongal* | Usili, Pulihora*, Vadiyam, Sambar, Rasam | Vadiyalu, Palam | Perugu Vada,  Annam with Pappu | Mr.Parvathi Kumar, 50/M | Telephonic conversation  29.11.20 | KA |
| 75 | Brahmin, Nambudri | Idali, Dosa, Pongal* Vadai* | Neichorru,Aviyal*, Varavu, Poricha*, Parappu Usili | Atirasam*,  Unni Appam* Laddu* | Nei Payasam*,  Dadhyodanam* | Mrs.Shubha, 52/F | Telephonic conversation  10.12.20 | KL |
| 76 | Brahmin, Vaishnava | Pongal* ,Vadai*, | Neyyannam, Pulihora*, Vadam, Avial, Sambar, Uroogayi, Rasam | Adirasam,  Guggillu* Laddu* Kova* | Dadhyodanam* Rice , Kolumbu, Rasavali | Mrs. Narayanan, 60/M | In-person  18.12.20 | TN |
| 77 | Brahmins, Badaganadu | Pongal*, Vada*, Upma, Idly, Dosa | Neyyannam, Pulihora*, Chitrannam*, Aravani, Paramannam, Vadiyam, Majjiiga, Pulusu, Gojju, Vepudu, Koora, Sambar, Uragaya, Rasam, Appadam | Aravani, Atirasam (only prepared during ancestral ceremonies)  Guggillu* Laddu* Kova* | Dadhyodanam* Tayir Vada, Rasavali | Mr.Veda Murthy, 70/M | In-person  22.12.20 | AP |
| 78 | Arya Vysya | Pongal* Vada*, Idly, Dosa, Avalakki | Tuppadanna, Pulihora*, Anna Sambar, Sandigae, Rasam | Adirasam (ancestral ceremonies)  Sundal* Laddu* Kova* | Dadhyodanam* Perugu Vada, Rice Rotti, Rice Rasam, Rasavali | Ms.Padmavathi, 75/F | In-person  24.12.20 | KA |
| 79 | Lingayatha, Shivagama | Pongal*, Vada*, Idly, Dosa | Rice, Pulihora*, Vadiyam, Sambar, Pappu, Rasam | Atirasam, Sundal*, Laddu* Kova* | Dadhyodanam* Perugu Vada, Rasavali | Mr. Umeswar, 53/M | In-person  26.12.20 | KA |
| 80 | Brahmin, Nambudri | Idly, Dosa, Pongal* Vada* | Neichorru, Aviyal*, Varavu, Poricha*, Pulippu Koottu, Parappu Usili | Atirasam*  Unni Appam* Laddu* | Pal Payasam*,  Dadhyodanam* | Mr.Varun, 69/M | Telephonic conversation  03.01.21 | KL |
| 81 | Brahmin, Vaishnava | Dosa, Idlali, Venn Pongal* Ulundu Vadai* | Palakaram, Adai, Sadam, Kolumbu, Avial Satramudu, Dadhyodanam* | Atirasam*, Rasavali, Sidai* | Sadam and Satramudu, Rasavali | Dr.Sowmya Narayanan, 60/F | In-person  12.01.21 | TN |
| 82 | Brahmins, Vaishnava | Dosa, Idly, Pongal*, Perugu Vada, | Usili, Pulihora*, Vadiyam, Palya  Sambar, Rasam | Vadiyalu, Palam | Palam with honey | Mrs. Kamakshi, 70/F | In-person  15.01.21 | KA |
| 83 | Brahmins, Smartha | Dosa, Idly, Pongal*, Vada*, Rasayanam* | Usili, Pulihora*, Vadiyam, Majjiigae Huli, Gojju, Palya, Sambar, Rasam | Guggillu*  Vadiyalu | Perugu Vada, Annam with Pappu, | Mr.Subrahmanya Shastry, 69/M | In-person  19.01.21 | KA |
| 84 | Brahmins, Niyogi | Pongal*, Vada*, Idly, Dosa | Neyyannam, Chitrannam*, Pulihora*, Vadiyam, Sambar, Uragaya, Rasam | Adirasam (ancestral ceremonies)  Guggillu* Laddu* Kova* | Dadhyodanam* Perugu Vada, Rasavali | Mrs.Vanishree, 51/F | Telephonic conversation  29.01.21 | TG |
| 85 | Brahmins, Badaganadu | Idly, Pongal*, Vada*, Dosa | Pulihora*, Chitrannam*, Vadiyam, Gojju, Palya, Sambar, Rasam | Kajjaya (ancestral ceremonies), Rasavali, | Dadhyodanam* Perugu Vada, Rice with Rasam | Mrs.Sumithra, 62/F | In-person  30.01.21 | AP |
| 86 | Arya Vysya | Pongal* ,Vada*,Idly, Dosa, Rotti | Neyyannam, Chitrannam*, Pulihora*, Vadiyam, Majjiiga, Pulusu, Gojju, Vepudu, Koora, Sambar, Uragaya, Rasam, Dadhyodanam* | Kajjaya (ancestral ceremonies)  Guggillu* Laddu* Kova* | Perugu Vada, Rasavali | Mr. Karthik Shetty, 59/M | In-person  13.02.21 | AP |
| 87 | Lingayatha, Shivagama | Idly, Pongal* Vada* | Pulihora*, Vadiyam, Majjiigae huli, Gojju, Sambar, Rasam | Kajjaya (ancestral ceremonies)  Guggillu* Laddu* | Dadhyodanam* Perugu Vada, | Mrs. Uma, 69/F | In-person  16.02.21 | KA |
| 88 | Brahmin, Nambudri | Idly, Dosa, Pongal* Vada* | Neichorru,Aviyal*, Varavu, Poricha*, | Atirasam* (ancestral ceremonies)  Unni Appam* Laddu* | Nei Payasam*,  Dadhyodanam* | Mrs.Parvathi Ammal, 70/F | Telephonic  20.02.21 | KL |
| 89 | Brahmin, Vaishnava | Dosa, Idly, Pongal*, Perugu Vada, | Usili, Pulihora*, Vadiyam, Palya  Sambar, Rasam | Vadiyalu, Palam, Sukkinunde* | Avalakki, Palam with honey | Ms. Sandhya, 69/F | Telephonic  22.02.21 | KA |
| 90 | Brahmins, Badaganadu | Idly, Pongal*, Vada*, Dosa | Pulihora*, Vadiyam, Gojju, Palya, Sambar, Rasam | Kajjaya (ancestral ceremonies), Rasavali, | Dadhyodanam* Perugu Vada, Rice with Rasam | Mr. Kameswara Rao, 72/F | In-person  26.02.21 | KA |
| 91 | Brahmins, Vaishnava | Pongal*, Vadai*, Sugiyan* | Anna, Sambar, pulihora*, paramannam*, Rasam, Dadhyodanam* Tayir Vada, | Aravani, Adirasam (only prepared during ancestral ceremonies)  Laddu* Kova* | Avalakki, ,Rasavali | Prof.Puvirajan, 42/M | Telephonic  02.03.21 | TN |
| 92 | Brahmins, Smartha | Dosa, Idly, Pongal*, Vada*. | Pulihora*, Aravani, Paramannam, Vadiyam, Majjiiga, Pulusu, Gojju, Vepudu, Koora, Sambar, Uragaya, Rasam, Appadam | Aravani, Ariselu, Undalu*,  Guggillu*, Laddu*, Kova* | Dadhyodanam*, Perugu Vada, Rasavali | Dr.Ramesh, 72/M | In-person  04.03.21 | KA |
| 93 | Brahmins, Niyogi | Pongal* Vada* | Neyyannam, Pulihora*, Vadiyam, Majjiiga, Pulusu, Gojju, Vepudu, Koora, Sambar, Uragaya, Rasam | Adirasam (ancestral ceremonies)  Guggillu* Laddu* Kova* | Dadhyodanam* Perugu Vada, Rasavali | Mrs.Praveena, 50/F | In-person  12.03.21 | TG |
| 94 | Brahmins, Badaganadu | Idly, Pongal*, Vada*, Dosa | Pulihora*, Vadiyam, Gojju, Palya, Sambar, Rasam | Kajjaya (ancestral ceremonies), Rasavali* | Dadhyodanam* Perugu Vada, Rice with Rasam | Mr.Krishna Rao, 73/M | Telephonic  15.03.21 | KA |
| 95 | Arya Vysya | Idly, Pongal* ,Vada*, Rice Rotti, Avalakki | Pulihora*, Vadiyam, Majjiiga, Pulusu, Gojju, Vepudu, Sambar, Rasam | Kajjaya (ancestral ceremonies)  Guggillu* Laddu* | Dadhyodanam* Perugu Vada, Pongal, Upma | Mrs. Gayathri Lakshmi, 65/F | In-person  19.03.21 | AP |
| 96 | Lingayatha, Shivagama | Idly, Pongal* Vada* | Pulihora*, Vadiyam, Majjiigae huli, Gojju, Sambar, Rasam | Kajjaya (ancestral ceremonies)  Guggillu* Laddu* | Dadhyodanam* Perugu Vada, | Mrs. Bharathi, 66/F | In-person  12.04.21 | KA |
| 97 | Brahmin, Nambudri | Idly, Dosa, Pongal* Vada* | Neichorru,Aviyal*, Varavu, Poricha*, Kolumbu, Pulisseri | Thaen Palankal*, Tirattupal*,  Unni Appam* Laddu* | Nei Payasam*,  Dadhyodanam* | Mr. Kannan, 70/M | In-person  18.05.21 | KL |
| 98 | Arya Vysya | Idly, Pongal*, Vada*, rotti, Upma, Avalakki | Chitrannam*, Pulihora*, Vadiyam, Majjiiga, Pulusu, Gojju, Vepudu, Koora, Sambar, Uragaya, Rasam | Adirasam (ancestral ceremonies)  Guggillu* Laddu* Kova* | Dadhyodanam* Perugu Vada, Rasavali | Mrs.Kanyaka, 55/F | In-person  22.06.21 | AP |
| 99 | Lingayatha, Shivagama | Idly, Pongal*, Dosa, Upma | Pulihora*, Vadiyam, Majjiigae huli, Gojju, Sambar, Rasam | Kajjaya (ancestral ceremonies)  Sandigae* Laddu* | Dadhyodanam* Perugu Vada, | Mrs. Gowri Devi, 69/F | In-person  23.06.22 | KA |
| 100 | Arya Vysya | Idly, Pongal* Dosa, Avalakki | Pulihora*, Aravani, Paramannam, Vadiyam, Majjiiga, Pulusu, Gojju, Vepudu, Koora, Sambar, Uragaya, Rasam, Appadam | Aravani*, Ariselu*, Undalu*,  Guggillu*, Laddu*, Kova* | Dadhyodanam*, Perugu Vada, Rasavali | Mr.Pavan Shetty, 45/M | Telephonic  04.07.21 | TG |
| 101 | Lingayatha, Shivagama | Idly, Pongal* Vada*, Idly | Pulihora*, Vadiyam, Majjiigae huli, Gojju, Sambar, Rasam | Kajjaya  Sundal* Laddu* | Dadhyodanam* Perugu Vada, | Mr. Lingeswar, 67/M | In-person  15.07.21 | KA |
| 102 | Brahmins, Niyogi | Idly, Pongal*, Vada*, Dosa | Pulihora*, Vadiyam, Majjiiga, Pulusu, Gojju, Vepudu, Sambar, Rasam | Kajjaya (ancestral ceremonies)  Guggillu*, Laddu*, Purnalu* | Dadhyodanam* Perugu Vada, | Dr. Kalyan Ram, 48/M | In-person  28.07.21 | TG |
| 103 | Brahmins, Vaishnava | Pongal*, Vadai*, Idlali, Dosa, Rasavali*, Perugu Vada* | Palakaram, Adai, Sadam, Kolumbu, Avial Satramudu, Dadhyodanam* | Aravani*, Tirukannamudu*,  Sundal, Laddu* | Sadam with Sambar and Rasam,  Dadhyodanam* | Mrs.Vijayanthi Mala, 66/F | Telephonic conversation  19.10.20 | TN |
| 104 | Brahmins, Smartha | Dosa, Idly, Pongal*, Vada*. | Pulihora*, Chitrannam*, Aravani*, Paramannam*, Vadiyam, Majjiiga, Pulusu, Gojju, Vepudu, Koora, Sambar, Uragaya, Rasam, Appadam | Aravani, Ariselu, Undalu*,  Guggillu*, Laddu*, Kova* | Dadhyodanam*, Perugu Vada, Rasavali* | Mrs.Ramani, 72/F | In-person  22.10.20 | AP |
| 105 | Brahmins, Niyogi | Pongal*, Vada*, idly, Dosa, Avalakki | Neyyannam*, Pulihora*, Vadiyam, Majjiiga, Pulusu, Gojju, Vepudu, Koora, Sambar, Uragaya, Rasam | Adirasam (ancestral ceremonies)  Guggillu* Laddu* Kova* | Dadhyodanam*,  Rasavali* | Mrs.Radhika, 59/F | In-person  18.11.20 | TG |
| 106 | Brahmins, Badaganadu | Idly, Pongal*, Vada*, Dosa, Perugu Vada | Pulihora*, Vadiyam, Gojju, Palya, Sambar, Rasam | Kajjaya (ancestral ceremonies), Rasavali, | Dadhyodanam* Rice with Rasam | Mr.Achyutha Rao, 70/M | In-person  22.11.20 | KA |
| 107 | Arya Vysya | Pongal* Vada*, Idly, Dosa, Avalakki | Tuppadanna, Pulihora*, Anna Sambar, Sandigae, Rasam | Adirasam (ancestral ceremonies)  Sundal* Laddu* Kova* | Dadhyodanam* Perugu Vada, Rice Rotti, Rice Rasam, Rasavali | Mr. Aswartha Narayana Shetty, 79/M | Telephonic conversation  29.11.20 | KA |
| 108 | Lingayatha, Shivagama | Dosa, Idly, Pongal* | Usili, Pulihora*, Vadiyam, Pulusu,  Sambar, Rasam | Vadiyalu, Palam | Perugu Vada,  Annam with Pappu | Mr.Pramod Kumar, 52/M | Telephonic conversation  29.11.20 | KA |
| 109 | Brahmin, Nambudri | Idaly, Dosa, Pongal* Vadai*, Tirukannamadai* | Neichorru, Aviyal*, Varavu, Poricha* | Atirasam* (ancestral ceremonies)  Unni Appam* Laddu* | Nei Payasam*,  Dadhyodanam* | Mrs.Usha, 50/F | Telephonic conversation  10.12.20 | KL |
| 110 | Brahmin, Vaishnava | Pongal*, Vadai*, Sugiyan*, Idali, Dosa | Anna, Sambar, pulihora*, paramannam*, Rasam, Dadhyodanam* Tayir Vada, | Aravani, Adirasam (only prepared during ancestral ceremonies)  Laddu* Kova* | Avalakki, ,Rasavali | Mrs. Shakunthalamma, 70/F | In-person  18.12.20 | TN |
| 111 | Brahmins, Badaganadu | Pongal* Vada* | Neyyannam*, Chitrannam*, Pulihora*, Aravani*, Paramannam*, Vadiyam, Majjiiga, Pulusu, Gojju, Vepudu, Koora, Sambar, Uragaya, Rasam, Appadam, Dadhyodanam* | Aravani, Adirasam (only prepared during ancestral ceremonies)  Guggillu* Laddu* Kova* | Upma, Avalakki, Pongal*, Rice Rasam, Rasavali* | Mr. Narasimha Murthy, 60/M | In-person  22.12.20 | AP |
| 112 | Arya Vysya | Pongal* Vada*, Idly, Dosa, Avalakki | Tuppadanna*, Pulihora*, Anna Sambar, Sandigae, Rasam | Adirasam (ancestral ceremonies)  Sundal* Laddu* Kova* | Dadhyodanam* Perugu Vada, Rice Rotti, Rice Rasam, Rasavali | Mrs. Lakshmi, 60/F | In-person  24.12.21 | KA |
| 113 | Lingayatha, Shivagama | Idly, Pongal* Vada*, Idly | Pulihora*, Vadiyam, Majjiigae huli, Gojju, Sambar, Rasam | Kajjaya  Sundal* Laddu* | Dadhyodanam* Perugu Vada, | Mr. Somanathayya, 70/M | In-person  26.12.21 | KA |
| 114 | Brahmin, Nambudri | Idly, Dosa, Pongal*, Vada*, Vadai, Idali | Neichorru,Aviyal*, Varavu, Poricha* | Atirasam*  Unni Appam* Laddu* | Nei Payasam*,  Dadhyodanam* | Mrs. Unni Krishnan, 68/F | Telephonic conversation  03.01.22 | KL |
| 115 | Brahmin, Vaishnava | Pongal*, Vada*, Idly, Dosa, Rasavali*, Perugu Vada* | Palakaram*, Adai*, Sadam, Kolumbu, Avial Satramudu, Dadhyodanam* | Aravani*, Adirasam*, Tirukannamudu*,  Sundal*, Laddu* | Sadam with Sambar and Rasam,  Dadhyodanam* | Mrs.Shanthammal, 70/F | In-person  12.01.22 | TN |
| 116 | Brahmins, Vaishnava | Dosa, Idly, Pongal* , vada* | Usili*, Pulihora*, Vadiyam, Huli,  Sambar, Rasam | Sundal*, Palam | Perugu Vada,  Annam with Pappu | Mrs. Kamakshamma, 69/F | In-person  15.01.22 | KA |
| 117 | Brahmins, Smartha | Dosa, Idly, Pongal* Vada* | Usili*, Pulihora*, Vadiyam, Majjiiga, Pulusu, Gojju, Vepudu, Sambar, Rasam | Guggillu*  Vadiyalu | Perugu Vada, Anna Sambar, Dadhyodanam* | Mr. Nanjunda Shastry, 70/M | In-person  19.01.22 | KA |
| 118 | Brahmins, Niyogi | Pongal*, Vada*, Dosa, Idly | Neyyannam, Pulihora*, Vadiyam, Sambar, Uragaya, Rasam | Adirasam (ancestral ceremonies)  Guggillu* Laddu* Kova* | Annam with Pappu and Dadhyodanam* | Mrs. Rukminamma, 70/F | Telephonic conversation  29.01.22 | TG |
| 119 | Brahmins, Badaganadu | Dosa, Idly, Pongal*, Vada*. | Pulihora*, Chitrannam*, Aravani, Paramannam, Vadiyam, Majjiiga, Pulusu, Gojju, Vepudu, Koora, Sambar, Uragaya, Rasam, Appadam | Aravani, Ariselu, Undalu*,  Guggillu*, Laddu*, Kova* | Annam with Rasam and Dadhyodanam*, | Mrs.Sunitha, 50/F | In-person  30.01.22 | AP |
| 120 | Arya Vysya | Pongal*, Vada*, Upma, Avalakki, Idly, dosa, Rotti | Neyyannam*, Pulihora*, Sandigae, Majjiiga Huli, Gojju, Sambar, Uppinakaya, Rasam | Atirasam (ancestral ceremonies)  Guggillu* Laddu* Kova* | Anna with Rasam, Rotti, Pongal, upma | Mr. Raghottam Shetty, 58/M | In-person  13.02.22 | KA |
| 121 | Lingayatha, Shivagama | Idly, Pongal* Vada*, Idly | Pulihora*, Vadiyam, Majjiigae huli, Gojju, Sambar, Rasam | Kajjaya *,  Sundal* Laddu* | Dadhyodanam* Anna Rasam | Mrs.Parameshwari, 55/F | In-person  16.02.22 | KA |
| 122 | Brahmin, Nambudri | Idly, Dosa, Pongal*, Vada*, Puttu, Appam*, Tayir Vadai | Pulihogarai, Neichorru*, Aviyal*, Varavu, Poricha*, Puliporiyal, Parippu, Kolambu*, Rasam | Atirasam*,  Unni Appam*, Varavu, Vatral, Sukiyan*, Tirattupal* | Pal Payasam*, Nei Chorru with Rasam,  Dadhyodanam* | Mrs.Jayaraman, 68/F | Telephonic  20.02.22 | KL |
