# Supplementary material for: Vegetarian ethnic foods of South India: review on the influence of traditional knowledge
Source: J. Ethn. Food. 2022 Oct 21;9(1):42. doi: 10.1186/s42779-022-00156-1 (PMC9589551; doi:10.1186/s42779-022-00156-1)
Supplement: Supplementary file 2 — Additional file 2: Appendix 2: Few photographs of Semi-structured interviews conducted at Temples (Photos taken only with the consent of the respondents) [file 42779_2022_156_MOESM2_ESM.docx]

**Appendix 2**

**Few photographs of Semi-structured interviews conducted at Temples**

**(Photos taken only with the consent of the respondents)**

**Few photographs of semi structured interviews and informal conversations with Brahmins, Arya Vysya, Lingayat and Namboodiri families**

**Academic works of First Author (Dr.Srinidhi.K.Parthasarathi) on contemporary applications of Indian Philosophy**

**
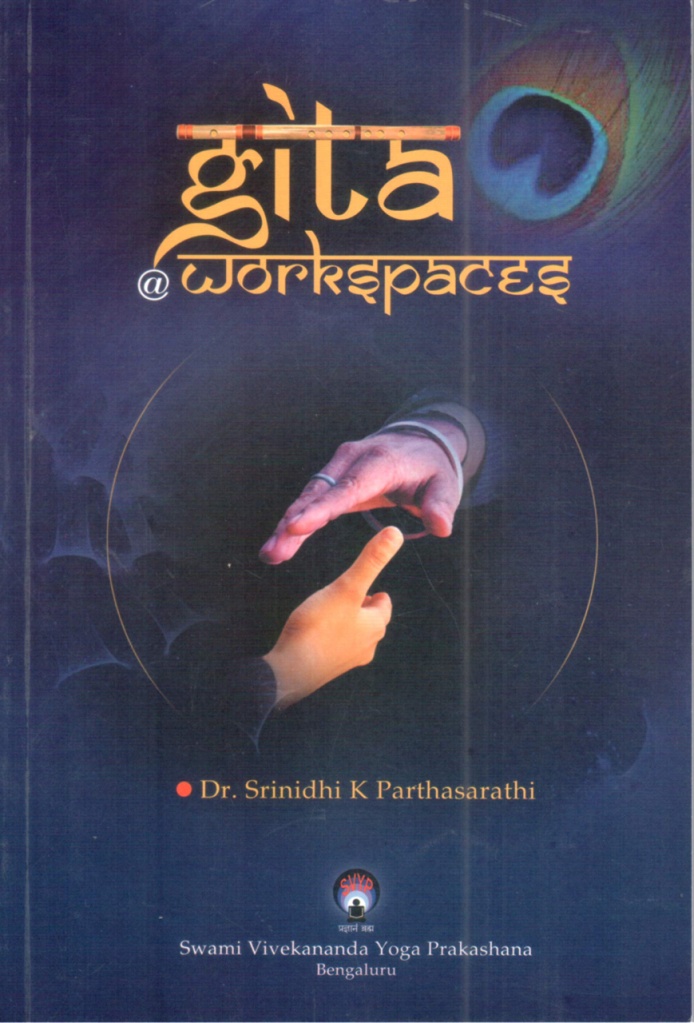
**

**Religious discourses by the First Author (Dr.Srinidhi.K.Parthasarathi) on topics of Indic traditional texts viz., Ramayana, Mahabharata, Divyaprabhandam**

**
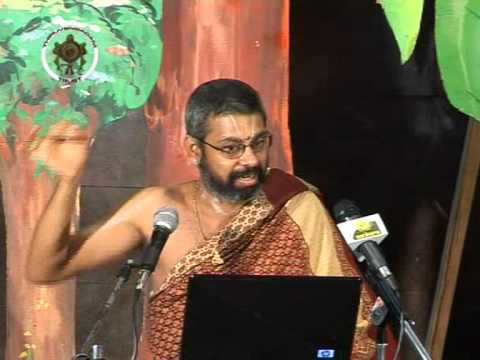

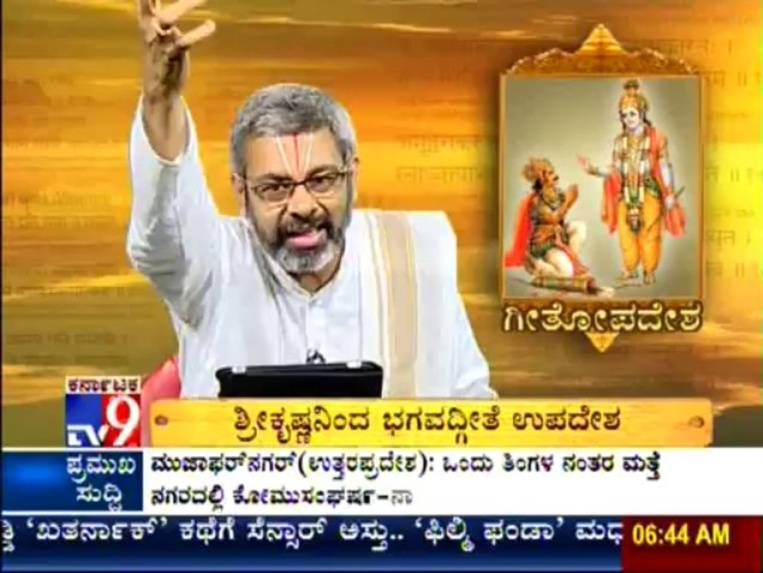
**

**
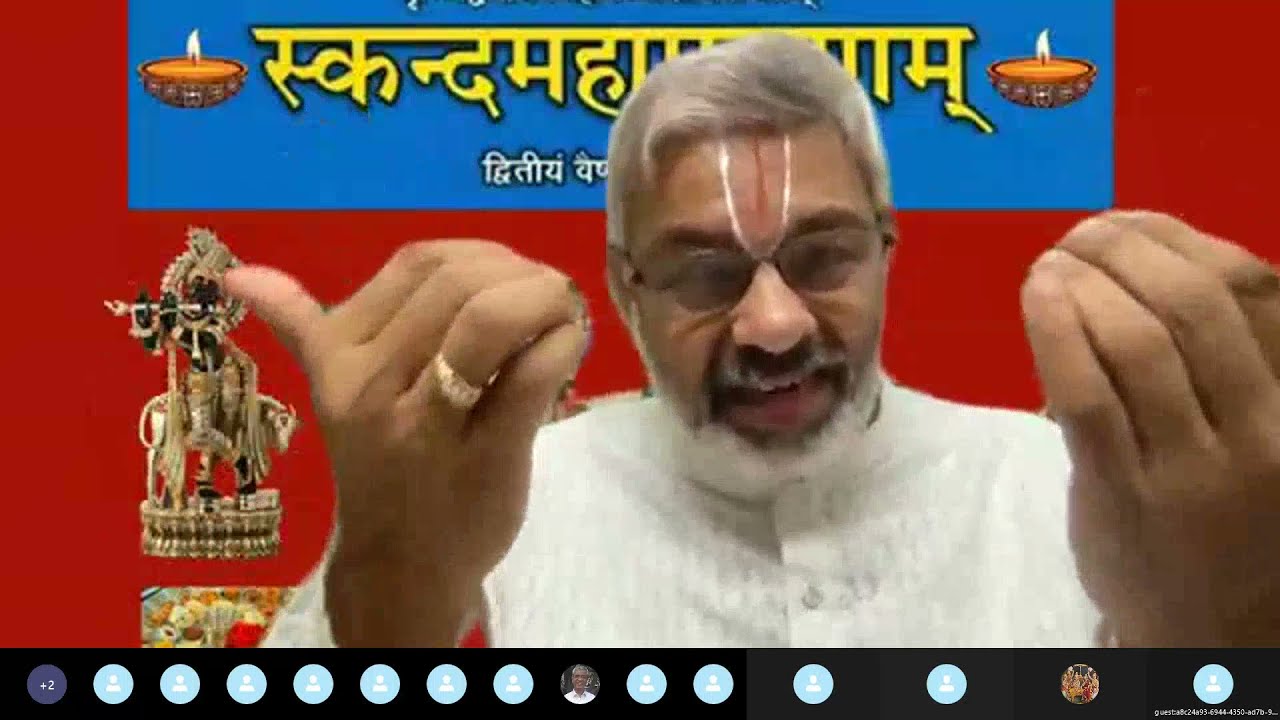

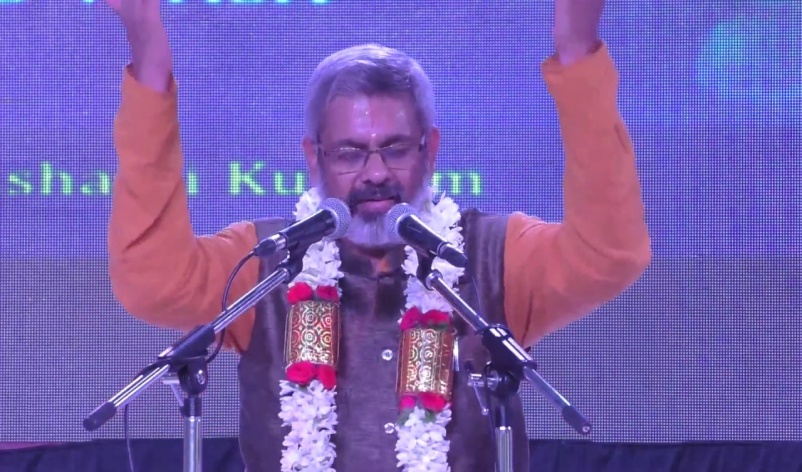
**

**Social media handles of the First Author (Dr.Srinidhi.K.Parthasarathi)**

[**https://indianacademy.edu.in/2021/09/20/dr-srinidhi-k-parthasarathi-2/**](https://indianacademy.edu.in/2021/09/20/dr-srinidhi-k-parthasarathi-2/)

[**https://www.linkedin.com/in/dr-srinidhi-k-parthasarathi-3951b432/?originalSubdomain=in**](https://www.linkedin.com/in/dr-srinidhi-k-parthasarathi-3951b432/?originalSubdomain=in)

[**https://www.sanskritfromhome.org/teacher-profile/Dr.-Srinidhi-K-Parthasarathi-58450216**](https://www.sanskritfromhome.org/teacher-profile/Dr.-Srinidhi-K-Parthasarathi-58450216)

[**https://www.facebook.com/DrSrinidhi-K-Parthasarathi-728693717305796/**](https://www.facebook.com/DrSrinidhi-K-Parthasarathi-728693717305796/)

[**https://www.youtube.com/channel/UCD_mgoSBouGSTnCFdaDuHwA/videos**](https://www.youtube.com/channel/UCD_mgoSBouGSTnCFdaDuHwA/videos)
